# Pericytes Regulate Vascular Basement Membrane Remodeling and Govern Neutrophil Extravasation during Inflammation

**DOI:** 10.1371/journal.pone.0045499

**Published:** 2012-09-21

**Authors:** Shijun Wang, Canhong Cao, Zhongming Chen, Vytas Bankaitis, Eleni Tzima, Nader Sheibani, Keith Burridge

**Affiliations:** 1 Lineberger Cancer Center, University of North Carolina, Chapel Hill, North Carolina, United States of America; 2 Department of Cell and Developmental Biology, University of North Carolina, Chapel Hill, North Carolina, United States of America; 3 Department of Cell and Molecular Physiology, University of North Carolina, Chapel Hill, North Carolina, United States of America; 4 McAllister Heart Institute, University of North Carolina, Chapel Hill, North Carolina, United States of America; 5 Department of Ophthalmology and Visual Sciences, University of Wisconsin School of Medicine and Public Health, Madison, Wisconsin, United States of America; Lerner Research Institute, United States of America

## Abstract

During inflammation polymorphonuclear neutrophils (PMNs) traverse venular walls, composed of the endothelium, pericyte sheath and vascular basement membrane. Compared to PMN transendothelial migration, little is known about how PMNs penetrate the latter barriers. Using mouse models and intravital microscopy, we show that migrating PMNs expand and use the low expression regions (LERs) of matrix proteins in the vascular basement membrane (BM) for their transmigration. Importantly, we demonstrate that this remodeling of LERs is accompanied by the opening of gaps between pericytes, a response that depends on PMN engagement with pericytes. Exploring how PMNs modulate pericyte behavior, we discovered that direct PMN-pericyte contacts induce relaxation rather than contraction of pericyte cytoskeletons, an unexpected response that is mediated by inhibition of the RhoA/ROCK signaling pathway in pericytes. Taking our *in vitro* results back into mouse models, we present evidence that pericyte relaxation contributes to the opening of the gaps between pericytes and to the enlargement of the LERs in the vascular BM, facilitating PMN extravasation. Our study demonstrates that pericytes can regulate PMN extravasation by controlling the size of pericyte gaps and thickness of LERs in venular walls. This raises the possibility that pericytes may be targeted in therapies aimed at regulating inflammation.

## Introduction

Inflammation is a host defense response to infection or injury. Triggered inappropriately, however, this reaction may induce progressive tissue injury, a clinical phenomenon that is involved in deterioration of inflammation-associated diseases such as progressive expansion of posttraumatic wounds and secondary tissue necrosis at sites of myocardial infarction [1], [2]. In addition, inappropriate inflammation underlies many inflammatory diseases, from rheumatoid arthritis and inflammatory bowel disease to asthma and multiple sclerosis. As key inflammatory cells, polymorphonuclear neutrophils (PMNs) participate in these pathological events by releasing bioactive products such as neutrophil elastase and oxygen free radicals into sites of inflammation [3], a process that requires circulating PMNs to be recruited out of the circulation and across venular walls, i.e. neutrophil extravasation. Migrating across venular walls, PMNs have to traverse two cellular layers, crossing first the endothelium and then the pericyte sheath, and their associated basement membrane (BM) before moving into the interstitium [4].

Whereas much is known about how PMNs breach the endothelium during extravasation, little is known about how migrating PMNs traverse the pericyte sheath and vascular BM [4], [5], [6]. However, migrating leukocytes tend to accumulate in the space between the endothelium and vascular BM suggesting that the latter presents a significant barrier to leukocyte transmigration [7], [8]. Regarding crossing the BM, it was previously shown by one of us (S. Wang) that PMNs preferentially penetrate special areas within the mouse cremaster vascular BM where matrix proteins (e.g. laminins and collagen type IV) are distributed sparsely. These regions, referred to as low expression regions (LERs), become enlarged and thinner in a process of inflammation-induced remodeling of the vascular BM [9]. The ubiquitous existence of LERs and inflammation-induced remodeling of these sites was further confirmed in multiple tissues by Voisin et al [10]. However, little is known about how remodeling of LERs occurs during acute inflammation. In these previous studies, pericytes were simply used as reference points to indicate the location of LERs in venular walls. While they demonstrated that LERs align with gaps between adjacent pericytes [9], [10], these studies did not address whether pericytes contribute to the remodeling of the vascular BM at the LERs, or investigate whether pericytes play a role in leukocyte transmigration.

Unlike endothelial cells (ECs), which line the interior surface of blood vessels as a continuous cell monolayer linked by cell-cell junctions, pericytes are arranged in a sheath surrounding the endothelium [4], [9], [11], [12]. No specialized intercellular junctions have been identified between pericytes [11]. Both ECs and pericytes contribute to the formation of the vascular BM. However, within venular walls, the EC monolayer contacts the vascular BM with its abluminal surface, whereas the pericyte sheath is embedded in this matrix [4], [9], [11], [13]. The location of pericytes and their morphology surrounding the endothelium, coupled with their capacity to contract or relax, imply that structural changes in the pericyte sheath may affect the organization of the vascular BM during inflammation and influence PMN transmigration.

Here we have examined the response of pericytes to PMNs in IL-1β-stimulated venular walls in mouse cremaster muscles. Our results demonstrate that during inflammation direct engagement between migrating PMNs and pericytes induces expansion of pericyte gaps and their associated LERs in the vascular BM, thereby facilitating PMN passage across inflamed venular walls. Exploring the engagement of activated PMNs with mouse primary pericytes in culture reveals that PMNs induce pericyte relaxation via inhibition of the RhoA/Rho Kinase (ROCK) pathway and suppression of actomyosin-based contractility. Our study shows that it is relaxation rather than contraction of pericytes that opens the gaps between them to facilitate PMN extravasation.

## Materials and Methods

### Ethics Statement

All experiments were approved by the Institutional Animal Care Committee of the University of North Carolina School of Medicine and Public health and performed according to the legislation for the protection of animals (IACUC ID: 12–038.0).

### Experimental Animals

Wild type (WT) C57BL/6 mice were purchased from the Jackson lab. Platelet-endothelial cell adhesion molecule–deficient (PECAM-1^−/−^) mice on a C57BL/6 background were kindly provided by Dr P. Newman (Blood Research Institute, Blood Center of Wisconsin, Milwaukee). C57BL/6 immorto-mice expressing a SV40 large T antigen were obtained from Charles River Laboratories (Wilmington, MA).

### Inflammatory Response in Mouse Cremaster Muscles or Ear Skin and Intravital Microscopy

WT or PECAM-1^−/−^ male mice (∼25 g) were injected intrascrotally (i.s.) with saline, interleukin-1β (IL-1β) (50 ng/mouse) or tumor necrosis factor-α (TNF-α) (500 ng/mouse) (R&D Systems, Minneapolis, MN). In IL-1β-injected mouse cremaster muscles, the responding leukocytes (>90%) were previously verified to be PMNs [14]. In selected experiments where PMNs need to be depleted, 100 µg of anti-GR1 antibodies (Abs) [15] (RB6–8C5, BD Biosciences, Chicago, IL) were injected intraperitoneally (i.p.) into each mouse 24 hours before i.s. administration of IL-1β. To block PMN extravasation, in some experiments each mouse was intra-splenically pre-injected with 100 µg of Abs against either mouse intercellular adhesion molecule-1 (ICAM-1) (YN1/1.7.4, Biolegend, San Diego, CA) [16] or α_6_ integrin (GoH3, Biolegend, San Diego, CA) [17], [18] 15 minutes before IL-1β administration. Control animals received isotype-matched control Abs (Biolegend, San Diego, CA). Two hours after IL-1β injection, the cremaster muscles of fully anaesthetized mice were exteriorized and transferred to the stage of an Olympus FluoView FV1000 upright microscope and the cremaster muscles were constantly superfused with warm buffered solution. The numbers of rolling or static adherent leukocytes were quantified starting from the administration of superfusion buffer as described before [14]. In some experiments, cremaster muscles were clamped using a micro aneurysm clip (Harvard Apparatus, Holliston, MA) and superfused with buffer containing Norepinephrine (NE, 10 µM, Sigma-Aldrich, Saint Louis, MO) or Tolazoline (100 µg/ml, Sigma-Aldrich, Saint Louis, MO). Fifteen min later, the inflammatory response was stopped immediately by perfusion of 4% paraformaldehyde (PF). The cremaster muscles of all experimental mice were finally harvested and fixed in PF for further immunostaining. In some experiments, IL-1β (5 ng/mouse) or TNF-α (50 ng/mouse) in 50 µl saline was subcutaneously injected into mouse ears for two hours to induce an inflammatory response in skin.

### Immunostaining of Mouse Cremaster Muscles and Ear Skin

Fixed mouse cremaster muscles and ear skin were immunostained for multiple proteins as described previously [9]. An Alexa 488- or 647-labeled rat anti-mouse PECAM-1 Ab (MEC13.3 BioLegend, San Diego, CA) was applied to label ECs. The vascular BM was displayed by a rabbit anti–mouse collagen IV polyclonal Ab (Abcam, Cambridge, MA) plus appropriate secondary Abs conjugated to Alexa 405 or 488 (Invitrogen, Carlsbad, CA). To mark the PMNs, a goat anti–mouse MRP-14 Ab (Calgranulin B (M-19), Santa Cruz Biotechnology, Santa Cruz, CA) or Allophycocyanin-conjugated rat anti-mouse CD11b Ab (M1/70, eBioscience, San Diego, CA) was used. To stain the pericytes, tissues were further incubated with a Cy3-labeled anti-mouse α-SMA Ab (1A4, Sigma-Aldrich, St. Louis, MO) or a goat IgG against mouse Platelet-derived growth factor receptor-β (PDGFR-β) (R&D Systems, Minneapolis, MN) plus fluorescent secondary Abs. In all studies, appropriate control IgG (BioLegend, San Diego, CA) was used in parallel with the specific primary Abs.

### Imaging and Analysis of Immunostained Cremaster Muscles and Ear Skin

Immunostained samples were imaged in three-dimensions (3D) using a Zeiss 510 Meta confocal microscope with lasers excited at 488, 545, 633, and 740 nm respectively, and with a x40 water-dipping objective (numerical aperture = 1.25). The imaged tissues were quantified for transmigrating leukocytes, defined as the number of leukocytes within the immunostained venular walls (per 200 µm vessel segment) and extravasated leukocytes, defined as the number of leukocytes in the interstitium of the whole tissues (per mm^2^ cremaster muscle) or in the interstitial tissues within 100 µm around a 200 µm vessel segment. 3D images of vessels were split in the middle along the longitudinal axis using the LSM software and fluorescence intensity of “hemi-vessels” was analyzed. The area of gaps between adjacent pericytes, the area of Collagen IV LERs, and the protein contents of Collagen IV in LERs (fluorescence intensity, pixels/unit area), were measured as previously described [9].

### Isolation of Mouse Peritoneal PMNs

Mouse PMNs were harvested from the thioglycollate-stimulated peritoneal cavity as previously described [19]. Briefly 1 ml of 4% thioglycollate solution (Sigma-Aldrich, St. Louis, MO) was injected into the mouse peritoneal cavity. Four hours later, exudate cells were harvested and purified for PMNs by Percoll density gradient centrifugation, yielding PMN preparations of around 90% purity.

### Collection of Mouse Bone Marrow PMNs and Culture of Mouse Retinal Pericytes

Bone marrow neutrophils were harvested from wash-outs of femurs of mice using three-layer Percoll gradients, yielding neutrophil preparations of around 85% purity [20]. Primary pericytes were isolated from retinas of WT Immorto mice (6–7 pups, 4 week old). Twelve to fourteen retinas were collected and digested at 37°C for 45 min in serum-free DMEM containing collagenase type II (1 mg/ml, Worthington, Lakewood, NJ) and 0.1% BSA. After washes, digested tissue was resuspended in DMEM supplied with 10% FBS, 2 mM L-glutamine, 100 g/ml streptomycin, 100 U/ml penicillin, and murine recombinant interferon-γ (at 44 U/ml, R&D Systems, Minneapolis, MN) and cultured at 33°C with 5% CO_2_. Cells were progressively passed to larger plates [21] and used for different assays within 20 passages.

### Collection of Transmigrated PMNs in vitro

Within a 6-well Boyden transwell, mouse primary retinal endothelial cells were seeded on the top surface of each FN-pre-coated insert (with 3 µm pores) and grown to confluence. Then the endothelial cell layer was incubated with serum-free DMEM containing TNF-α (10 ng/ml) overnight and freshly isolated mouse bone marrow PMNs were added on the top of the endothelial cells. The inserts were put back into the wells containing serum-free DMEM in the presence of 3.0×10^−8^ M of interleukin-8 (IL-8, R&D Systems, Minneapolis, MN) and incubated at 37°C for 4 hours. Transmigrated PMNs were harvested and washed for further experiments.

### Retroviral Packing and Infection

Retroviral packaging and infection were performed as previously described [22]. Briefly, human 293 FT cells (Invitrogen, Carlsbad, CA) were placed on poly (L-Lysine)-pre-coated dish and transfected with the packing plasmids pRSV-Rev, pMDLg/pRRE, pCMV-VSV-G (Addgene, Cambridge, MA) and the pLentiLox 5.0 shuttle plasmid encoding WT, constitutively active (CA) Q63L, or dominant negative (DN) T19N human RhoA. Virus-containing medium, together with 6 µg/ml of polybrene, was added to pericytes which were plated at 50% confluence. Forty eight hours later, protein expression was checked by using fluorescence microscopy and western blot.

### Plasmids and shRNA Construction

To generate a lentiviral construct encoding short-hairpin RNA (shRNA) specific to mouse RhoA, forward hairpin oligos 5′-[Phos] TGTCAAGCATTTCTGTCCAATTCAAGAGATTGGACAGAAATGCTTGACTTTTTTC -3′ and reverse oligos 5′-[Phos] TCGAGAAAAAAGTCAAGCATTTCTGTCCAATCTCTTGAATTGGACAGAAATGCTTGACA -3′ were annealed and ligated into the *Hpa*I and *Xho*I sites of the pLentiLox5.0 (pLL5.0) vector (Addgene, Cambridge, MA). The dual cassette vector (pLL5.0-shRNA) co-expresses enhanced green fluorescent protein (EGFP) as a reporter gene and shRNA targeting mouse RhoA under the control of CMV and the U6 promoter, respectively. For expression of exogenous RhoA, plasmids encoding human RhoA (GenBank Accession number: NM_001664), were directly constructed into the SacII and BamHI sites of the pLL5.0 vector with an EGFP reporter under the control of CMV. All plasmid constructions were verified by sequencing.

### RhoA Activity Assays

RhoA activity assays were performed as described previously [23]. Primary pericytes or cells expressing GFP vector or GFP-tagged WT, CA or DN RhoA were cultured in serum free medium containing TNF-α (15 ng/ml). Next day, cells were stimulated at 37°C for 1 hour by serum-free DMEM containing DMSO, Forskolin (100 µM, Ascent Scientific, Princeton, NJ) or lysophosphatidic acid (LPA, Enzo Life Sciences, Plymouth Meeting, PA) (1 µg/ml)) or mouse PMNs, which were pretreated with DMSO or phorbol 12-myristate 13-acetate (PMA, Sigma-Aldrich, Saint Louis, MO) (100 ng/ml) and washed for four times with PBS. Cells were lysed and incubated with glutathione S-transferase (GST)-Rho binding domain (RBD) beads. Samples were washed and then resolved by SDS-page gels. The endogenous RhoA in the bound fraction (active) and in the whole cell lysates (total) was immune-blotted with an anti-RhoA Ab (26C4, Santa Cruz Biotechnology, CA). The exogenous active or total RhoA was detected with an anti-GFP monoclonal Ab (Roche Applied Science, Indianapolis, IN). The results were quantified using Image J software. The relative amount of active RhoA was determined by taking the ratio of RhoA sedimented by GST-RBD beads to the amount of total RhoA.

### Impedance-based Assay of Pericyte Responses to Either Reagents or PMNs

Mouse primary pericytes were grown in serum free medium containing TNF-α (15 ng/ml). Next day, 4.0×10^4^ cells were seeded onto gold electrodes in each well (surface area of each well is equal to 19.2 mm^2^/well) of a RTCA analyzer (Roche Applied Science, Indianapolis, IN) and electrical impedance was monitored over time. Changes in impedance may be used to measure a number of characteristics of cells in a real-time manner including cell spreading, degree of cell confluence, cell invasion, and permeability of a cell layer, depending on the experimental design (https://www.roche-applied-science.com/sis/xcelligence/index.jsp?&id=xcect_020000). Here we use the RTCA analyzer to monitor the spreading and morphology of pericytes within a confluent culture and their response to a number of factors. To verify our experimental results collected by the RTCA analyzer, in some selected experiments, changes of cell morphology at indicated time-points were analyzed in parallel, by imaging techniques as detailed below. In an equivalent experiment using 96-well plates, we found that 4.0×10^4^ pericytes were able to make a confluent cell monolayer and cover a surface of 19.2 mm^2^ 2 hours after spreading. Our preliminary experiments indicated that the impedance for each well reached a plateau from 2 to 6 hours after cell seeding. Within this time frame, reagents such as DMSO, PBS, Forskolin (100 µM), C3 Transferase (C3) (2 µg/ml, Cytoskeleton Inc, Denver, CO), Y27632 (10 µM, Calbiochem, Billerica, MA), Blebbistatin (10 µM, Sigma-Aldrich, Saint Louis, MO), Tolazoline (100 µg/ml), NE (10 µM), or LPA (1 µg/ml), or 5.0×10^4^ mouse PMNs, were added to the pericyte layers and the impedance was recorded in real-time. In the case of adding PMNs, these cells were pretreated with DMSO, saline, PMA (100 ng/ml), or MnCl_2_ (2 mM) and extensively washed using 10 ml PBS at least four times before adding to the pericyte monolayers.

### Image-based Analysis of Pericyte Responses to Reagents or PMNs

Coverslips (12 mm in diameter) placed in the wells of a 24 well plate were pre-coated with human Fibronectin (FN, 10 µg/ml). Mouse pericytes (primary cells or cells expressing GFP vector or GFP-tagged WT, CA or DN RhoA) were grown in serum free medium containing TNF-α (15 ng/ml). On the next day cells were seeded onto each well at 37°C for 4 h, allowing cells to fully spread. During this time period, PMNs were freshly isolated from mouse bone marrow and treated with DMSO, saline, PMA (100 ng/ml), MnCl_2_ (2 mM) or recombinant murine CXCL1 (KC) (1×10^−7^ M) at 37°C for 15 min. After being extensively washed, 5.0×10^5^ pretreated PMNs were added onto the pericyte layer at 37°C for 1 hour and cells were fixed in 4% PF. Alternatively, chemicals such as DMSO, PBS, Forskolin (100 µM), C3 (2 µg/ml), Y27632 (10 µM), Blebbistatin (10 µM), Tolazoline (100 µg/ml), NE (10 µM), or LPA (1 µg/ml) were added at 37°C for 1 hour to the cultures. In some selected experiments, pericytes were co-incubated for 1 hour with mouse peritoneal PMNs or PMNs which had passed across an endothelial cell layer and migrated to IL-8 in the bottom well of a Boyden chamber transwell system. Fixed cells were stained with Alexa-labeled phalloidin (Invitrogen, Carlsbad, CA), Hoechst 33342 (Invitrogen, Carlsbad, CA) and primary Abs against either paxillin (rabbit anti-paxillin Abs, Abcam, Cambridge, MA) or phosphorylated myosin light chain 2 (ser19) (anti-phospho-MLC Abs, Cell Signaling Technology, Danvers, MA) plus appropriate fluorescent second Abs and imaged using a Zeiss 510 Meta multi-photon confocal microscopy with lasers excited at 488, 545, 633 and 740 nm respectively, using a x63 oil objective (numerical aperture = 1.4). To view the behavior of pericytes, DMSO- or PMA-pretreated PMNs were added onto a TNF-α-induced pericyte layer, which had been grown on a FN-coated glass surface of culture dishes (MatTeK, Ashland, MA), and the interaction of pericytes and PMNs was recorded with a 20 x A-Plan ph1 objective and an ORCA ER CCD digital camera within a Zeiss Axiovert 200 M inverted microscope equipped with a heating stage and a CO2 supply and driven by a MetaMorph 7.1.7 software (MDS Analytical Technologies, Exton, PA).

### Measurement of the Area of Gaps between Adjacent Pericytes Grown in vitro

3.0×10^5^ mouse pericytes (primary cells or cells expressing GFP vector or GFP-tagged WT, CA or DN RhoA) in serum free medium containing TNF-α were seeded on FN-coated coverslips (12 mm in diameter) placed in the wells of a 24 well plate overnight. At this density, pericytes became confluent next day and were labeled with Calcein green. 5.0×10^5^ DMSO- or PMA-pretreated PMNs were labeled by Calcein blue and added on the top of each pericyte monolayer for 1 hour. Alternatively, compounds such as DMSO, Forskolin (100 µM), Tolazoline (100 µg/ml), NE (10 µM), or LPA (1 µg/ml) were added. Cells were fixed in 4% PF and 15 random fields per sample were imaged using a 20 x A-Plan ph1 objective in a Zeiss Axiovert 200 M microscopy using the setting for phase contrast or fluorescent images. The area of pericyte gaps (the area of negative staining in terms of fluorescence intensity) was measured using Image J software.

### Transmigration of PMNs Through a Pericyte Single Layer

Within Boyden transwells, 4.0×10^4^ pericytes (primary cells or cells expressing GFP vector or GFP-tagged WT or CA RhoA), which were grown in serum free medium containing TNF-α overnight, were seeded on the top surface of each FN-pre-coated insert (with 3 µm pores). Three hours later, cells in the inserts were stimulated with DMSO, Forskolin (100 µM), NE (10 µM), or NE (10 µM) plus Tolazoline (100 µg/ml) at 37°C for 1 hour and 2.0×10^5^ of Calcein-green labeled resting PMNs were added into each unit. Alternatively, pericytes were stimulated with 1.0×10^5^ DMSO- or PMA-pretreated PMNs for 1 hour followed by gentle washes with PBS and then 2.0×10^5^ of Calcein-green labeled resting PMNs were loaded into each insert. The inserts were put back into the transwells which were filled with serum-free RP1640 medium containing 3 10^−8^ M of IL-8 and incubated at 37°C for 4 h. Fluorescent PMNs which moved into the bottom wells were counted.

### Statistics

All results are expressed as means ± s.e.m. Statistical significance was assessed by one-way analysis of variance (ANOVA) plus Neuman-Keuls multiple comparisons. Where two variables were analyzed a Student’s t test was used. P<0.05 was considered to be significant.

## Results

### Inflamed Venules Exhibit Expanded Pericyte Gaps and Display Larger but Thinner LERs

**Figure 1:**
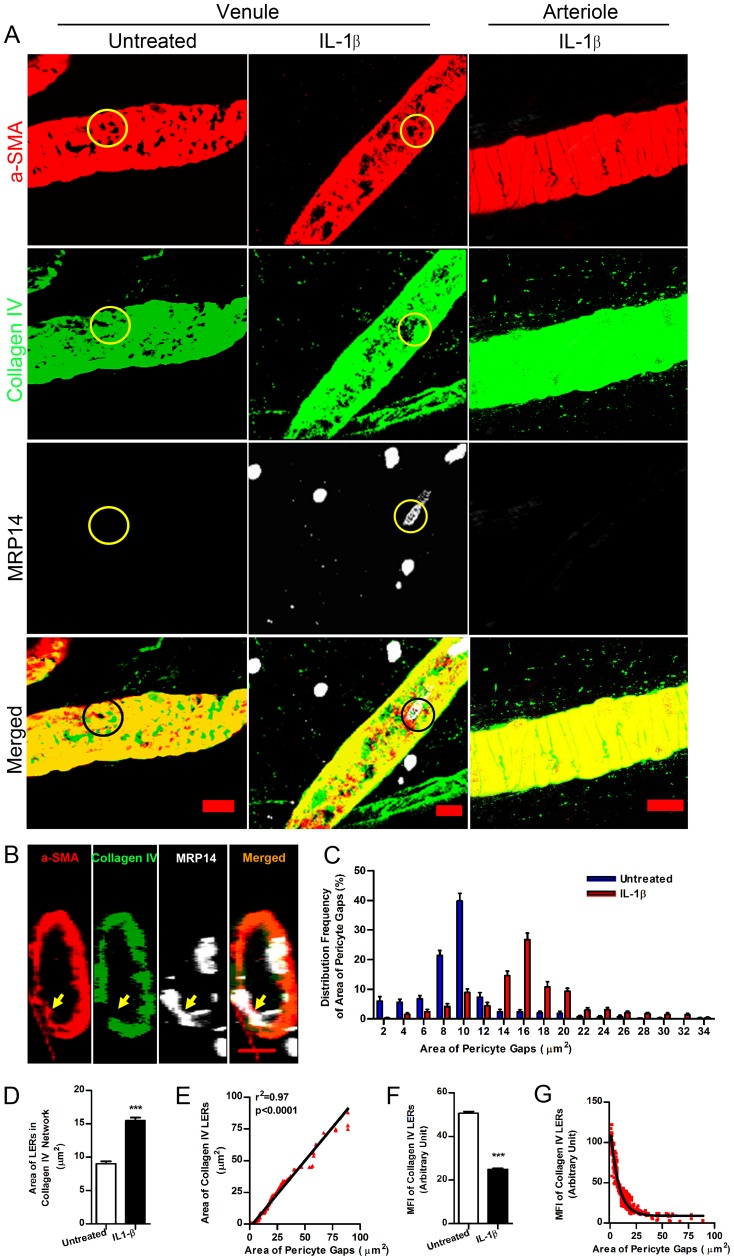
Expansion of pericyte gaps and collagen IV LERs in IL-1β-stimulated venular walls. (**A**) 3D images of vessel walls in mouse cremaster muscles in the presence and absence of IL-1β revealed pericyte gaps and collagen IV LERs (circled areas) in venules. α-SMA (a marker of venule pericytes and arteriole smooth muscles), collagen IV (a vascular BM marker) and MRP14 (a PMN marker). (**B**) Cross-sectional images revealed PMNs migrating through pericyte gaps and collagen IV LERs (arrows) in an IL-1β-stimulated venule. (**C**) Distribution frequency of area of pericyte gaps showed pericyte gap expansion induced by IL-1β (n = 2400). (**D**) Collagen IV LERs expanded in IL-1β-stimulated venular walls, compared to those in untreated vessels (In each group, n = 1500). (**E**) The area of pericyte gaps (n = 1500) was plotted against the area of collagen IV LERs (n = 1500). (**F**) Mean fluorescence intensity (MFI) of collagen IV in LERs decreased after IL-1β injection, indicating thinning of the vascular BM in these areas. (**G**) MFI of collagen IV in LERs was plotted against the size of pericyte gaps. Bar = 10 µm. t test, ***P<0.001.

Previous studies have suggested that the low expression regions (LERs) of matrix proteins in the vascular basement membrane (BM) act as channels for leukocyte transmigration and tend to align with the gaps between pericytes. These LERs are plastic with respect to their size and thickness during acute inflammation [9], [10]. However, little is known about how this phenomenon occurs and whether pericytes contribute to the remodeling of the vascular BM at these sites. To address this question, we injected mouse cremaster muscles or skin with IL-1β or TNF-α to elicit local acute inflammation. Two hours after injection, inflamed tissues were fluorescently immunostained and imaged by confocal microscopy. The area of gaps between adjacent pericytes, indicated by negative staining of α-SMA (or PDGFR-β, data not shown), and the area of LERs in the collagen type IV network were measured. Our data showed that pericyte gaps were enlarged after IL-1β injection (16.98±0.43 µm^2^), compared to those in untreated venules (9.70±0.30 µm^2^) (**Figure 1A, 1C**). PMNs used these gaps for transmigration (**Figure 1A, 1B**), which primarily occur at post-capillary venules rather than capillaries (**Figure S1**). In contrast, smooth muscle cells (SMCs), which share a common origin with pericytes in venules [11], revealed compact coil-like cell sheaths regularly surrounding the arterial vessels walls and few gaps between SMCs could be found (**Figure S2**). Moreover we were almost unable to detect PMN extravasation occurring in arterioles during acute inflammation (**Figure 1A** and **Figure S1**). This observation suggests that the occurrence of PMN extravasation at venules relies, at least in part, on the existence of gaps between adjacent pericytes in venular walls. Consistent with previous studies, IL-1β-stimulated LERs, as judged by collagen type IV immunofluorescence, expanded in area (**Figure 1A, 1D**) and became thinner (**Figure 1F**). Importantly, our data revealed a positive linear relationship between the area of pericyte gaps and collagen IV LERs but a negative correlation with LER thickness (**Figure 1E, 1G**). This is consistent with the expansion of pericyte gaps contributing to the remodeling of the vascular BM as LERs enlarge during acute inflammation.

### PMN Transmigration Expands Pericyte Gaps and LERs

**Figure 2:**
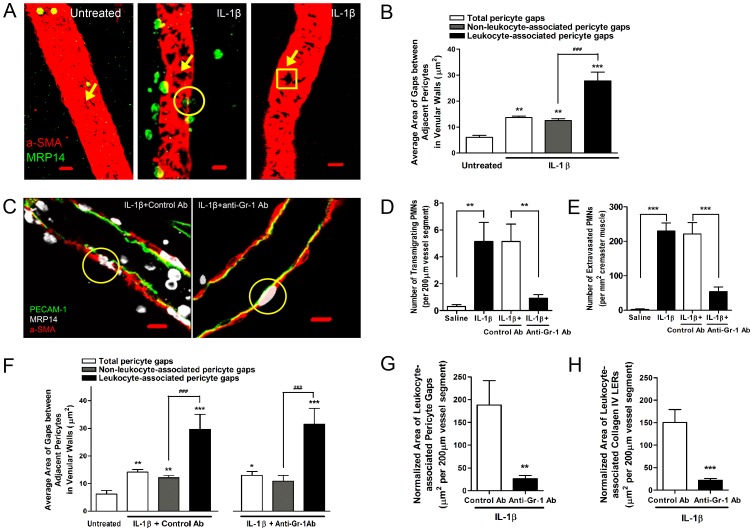
Depletion of circulating PMNs decreases the expansion of pericyte gaps and collagen IV LERs during inflammation. (**A**) 3D images showing the association of pericyte gaps and PMNs in mouse cremaster muscles that were untreated (left) or treated with IL-1β (middle and right). Arrows indicated pericyte gaps and a circle showed PMNs migrating through pericyte gaps. In IL-1β-stimulated tissues, two types of venular segments were noted. In some vessel segments (middle), both transmigrating and extravasated PMNs were seen. In others, no PMNs were found in the vessel walls or within 100 µm of the perivascular tissues (right). In the first situation (middle), pericyte gaps being used by transmigrating PMNs (circled area) are referred to as “leukocyte-associated pericyte gaps”. In the second situation (right, boxed area), pericyte gaps are referred to as “non-leukocyte-associated pericyte gaps”. Note: we did not classify the pericyte gaps not circled in the middle panel since we were not sure whether these gaps were used by leukocytes or not. However, these were included in the measurements when analyzing the total pericyte gaps. Similarly, collagen IV LERs were described as “leukocyte-associated LERs” or “non-leukocyte-associated LERs”. (**B**) Average area of pericyte gaps in untreated or IL-1β-stimulated mouse cremaster muscle venules. (**C**) 2D images along the longitudinal axis of venules treated with IL-1β together with control or anti-Gr-1 antibodies. Transmigrating PMNs are circled. (**D**) Number of transmigrating PMNs within venular walls treated with saline or IL-1β together with control or anti-Gr-1 antibodies. (**E**) Number of extravasated PMNs within mouse cremaster muscles treated with saline or IL-1β together with control or anti-Gr-1 antibodies. (**F**) Average area of pericyte gaps in untreated venules or vessels treated with IL-1β together with control or anti-Gr-1 antibodies. (**G**) The total area of leukocyte-associated pericyte gaps was normalized relative to a 200 µm length of vessel. (**H**) The total area of leukocyte-associated collagen IV LERs was normalized relative to a 200 µm length of vessel. Bar = 10 µm. 5–6 mice were used in each treatment. In (**B**) and (**F**), ANOVA plus Neuman-Keuls multiple comparisons were performed and in others, t tests were applied. *P<0.05, **P<0.01, and ***P<0.001, compared to untreated groups. ^###^P<0.001, compared between gaps as indicated.

Since migrating leukocytes prefer to use pericyte gaps and LERs in the vascular BM [9] and the expansion of pericyte gaps contributes to the enlargement of LERs during acute inflammation, we asked whether PMN transmigration might regulate the opening of pericyte gaps and hence the ensuing enlargement of LERs. To examine this, we fluorescently immunostained resting or IL-1β-injected mouse cremaster muscles and analyzed them using confocal microscopy. Normally few PMNs were observed in the lumen of resting venules (left panel in **Figure 2A**). Analyzing the association of PMNs and vessel walls, we noted two types of venular segments in IL-1β-stimulated tissues. In some segments transmigrating and fully extravasated PMNs were seen (middle panel in **Figure 2A**), whereas in others neither transmigrating nor extravasated PMNs were observed in the venular walls or in the perivascular tissues within 100 µm of these vessel segments (right panel in **Figure 2A**). In the former type of vessel segments, we refer to the pericyte gaps being used by transmigrating leukocytes, as leukocyte-associated pericyte gaps (circled in the middle panel in **Figure 2A**). In the latter, we refer to them as non-leukocyte-associated pericyte gaps (boxed in the right panel in **Figure 2A**). Accordingly, we refer to LERs as leukocyte-associated or non-leukocyte-associated collagen IV LERs (**Figure 1A** **)**. We found that the average area of pericyte gaps in untreated venules was less than that of non-leukocyte-associated gaps in IL-1β-stimulated vessels (**Figure 2B**), implying a general expansion of pericyte gaps occurs during inflammation induced by this cytokine. However, leukocyte-associated pericyte gaps were even larger than those non-leukocyte-associated (**Figure 2B**), suggesting that the interaction of pericytes with PMNs had expanded pericyte gaps. To examine this possibility, we depleted most (>89%) of circulating leukocytes using anti-Gr-1 antibodies [15]. This decreased the number of both transmigrating and extravasated PMNs in inflamed tissues (**Figure 2C–2E**). Since not all circulating PMNs can be removed by Gr-1 antibodies, in situations where transmigration occurred in anti-Gr-1 antibody-pretreated mice (right panel in **Figure 2C**), the leukocyte-associated pericyte gaps still had an average area similar to that found in animals pretreated with control antibodies (**Figure 2F**). However, when the total area of pericyte gaps and collagen IV LERs was normalized to a 200 µm vessel segment, leukocyte-associated pericyte gaps and collagen IV LERs in Gr-1 antibody-treated mice were much smaller than in control mice (**Figure 2G, 2H**). These experiments indicate that the presence of transmigrating PMNs further expands pericyte gaps as well as the LERs.

### Direct PMN-pericyte Contact Mediates Enlargement of Pericyte Gaps and LERs

**Figure 3:**
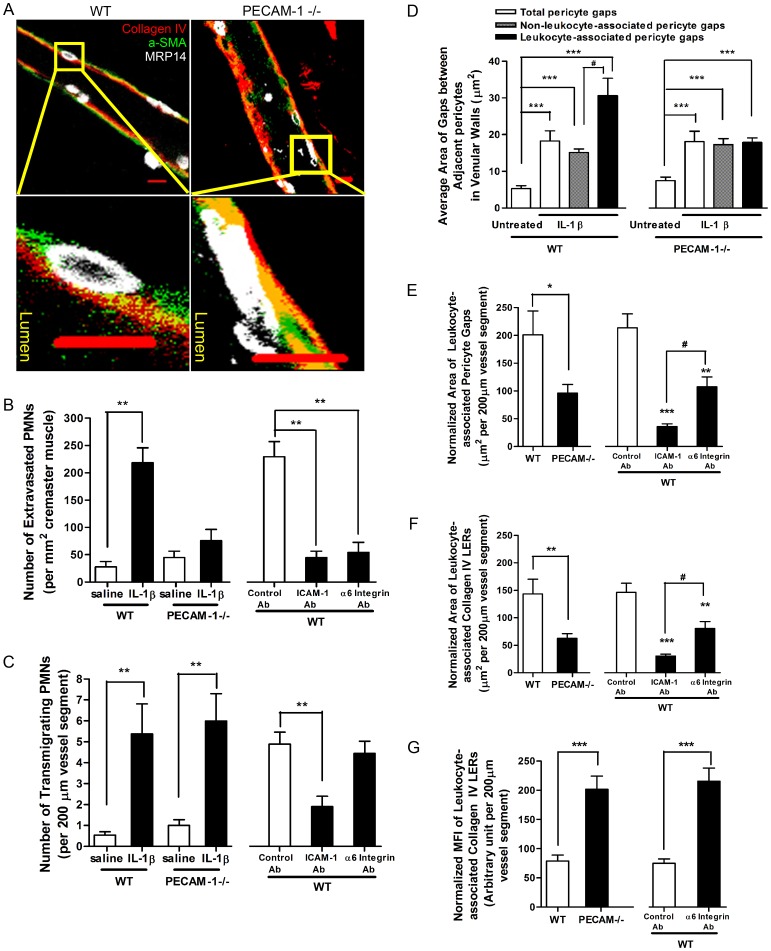
Direct cell-cell contact between transmigrating PMNs and pericytes mediates expansion of pericyte gaps and collagen IV **LERs.** (**A**) 2D images of IL-1β-stimulated venules along the longitudinal axis. The areas outlined in the top panels are shown magnified in the bottom panels, and reveal that transmigrating PECAM-1^−/−^ PMNs become trapped at the vascular BM. (**B**) Number of extravasated PMNs in WT or PECAM-1^−/−^ mouse cremaster muscles treated with saline or IL-1β, or in WT tissues injected with IL-1β together with control, anti-ICAM-1 or anti-α_6_ integrin antibodies. (**C**) Number of transmigrating PMNs within WT or PECAM-1^−/−^ venular walls treated with saline or IL-1β, or within WT vessels treated with IL-1β together with control, anti-ICAM-1 or anti-α6 integrin antibodies. (**D**) Average area of pericyte gaps in untreated or IL-1β-injected WT or PECAM-1^−/−^ venules in mouse cremaster muscles. (**E**) The area of leukocyte-associated pericyte gaps was compared for WT and PECAM-1^−/−^cremaster muscles, as well as with those treated with the indicated antibodies. (**F**) The area of leukocyte-associated collagen IV LERs was compared for WT and PECAM-1^−/−^cremaster muscles, as well as with those treated with the indicated antibodies. (**G**) The normalized MFI of leukocyte-associated collagen IV LERs was compared for WT and PECAM-1^−/−^ cremaster vessels, as well as with those treated with the indicated antibodies. Bar = 10 µm. 7–8 mice for each treatment. In panel (**D**), (**E**) and (**F**), ANOVA plus multiple comparisons (Comparisons with untreated (**D**) or control Ab (**E, F**) groups were displayed by *. Additional comparisons between two different groups were indicated by #), and in others, t tests were performed. * or ^#^P<0.05, **P<0.01, and ***P<0.001.

We next asked how migrating PMNs regulated the opening of pericyte gaps. The pericyte response to leukocytes could be mediated either by diffusible mediators or direct cell-cell contacts [11], [24]. Here we examined the role of direct contact between PMNs and pericytes. We took advantage of the observation that PMNs from PECAM-1^−/−^ mice display retarded transit across the IL-1β-stimulated venular BM within the first 4 hour time frame after IL-1β injection due to their failure to express α_6_ integrin on their cell surface [17], [20]. Hence in our study, we injected IL-1β into the cremaster muscles of wild type (WT) or PECAM-1^−/−^ mice to induce inflammation and used them as tools to test whether abolition of PMN-pericyte contact influenced the opening of pericyte gaps. In IL-1β-injected PECAM-1^−/−^ mice, the number of PMNs completing extravasation was significantly lower than in WT mice (**Figure 3A, 3B**), despite no difference in the number of PMNs involved in adhesion (observed by intravital microscopy) or attempting transmigration (**Figure 3C**). Migrating PECAM-1^−/−^ PMNs might pass across the endothelium but frequently stopped at the vascular BM, being separated from the pericyte sheath by this barrier (bottom right in **Figure 3A**), whereas WT PMNs broke through the vascular BM and reached the pericyte sheath (bottom left in **Figure 3A**). This measurement was previously confirmed by transmission electron microscopy [17]. The average area of total pericyte gaps and non-leukocyte-associated pericyte gaps in IL-1β-stimulated PECAM-1^−/−^ venules was greater than that in resting vessels, suggesting that a general expansion of pericyte gaps also occurs in PECAM-1^−/−^ venular walls during acute inflammation. Unlike WT PMNs, however, migrating PECAM-1^−/−^ PMNs did not cause any additional expansion of leukocyte-associated pericyte gaps (**Figure 3D**). These observations indicate that transmigrating PMNs separated from the pericyte sheath fail to enlarge pericyte gaps beyond the level induced by IL-1β.

Using intravital microscopy, we noted that anti-ICAM-1 antibodies dramatically inhibited leukocyte adhesion to the endothelium (data not shown). Immunostaining of whole-mounted tissues revealed that these antibodies reduced both transmigrating and extravasated PMNs in IL-1β-stimulated WT cremaster muscles (**Figure 3B, 3C**). Anti-α_6_ integrin antibodies did not influence the number of transmigrating PMNs in WT mice (**Figure 3C**) but trapped these leukocytes in the vascular BM (data not shown), therefore decreasing the number of emigrated PMNs (**Figure 3B**). Importantly, we found that both these antibodies markedly prevented expansion of both the leukocyte-associated pericyte gaps and the LERs (**Figure 3E, 3F**), similar to what was observed in PECAM-1^−/−^ animals (**Figure 3E–3G**). Taken together, these findings suggest that blocking contact between PMNs and pericytes prevents the signaling in pericytes required to expand the gaps between them.

### Engagement with Activated PMNs Induces Pericyte Relaxation

**Figure 4:**
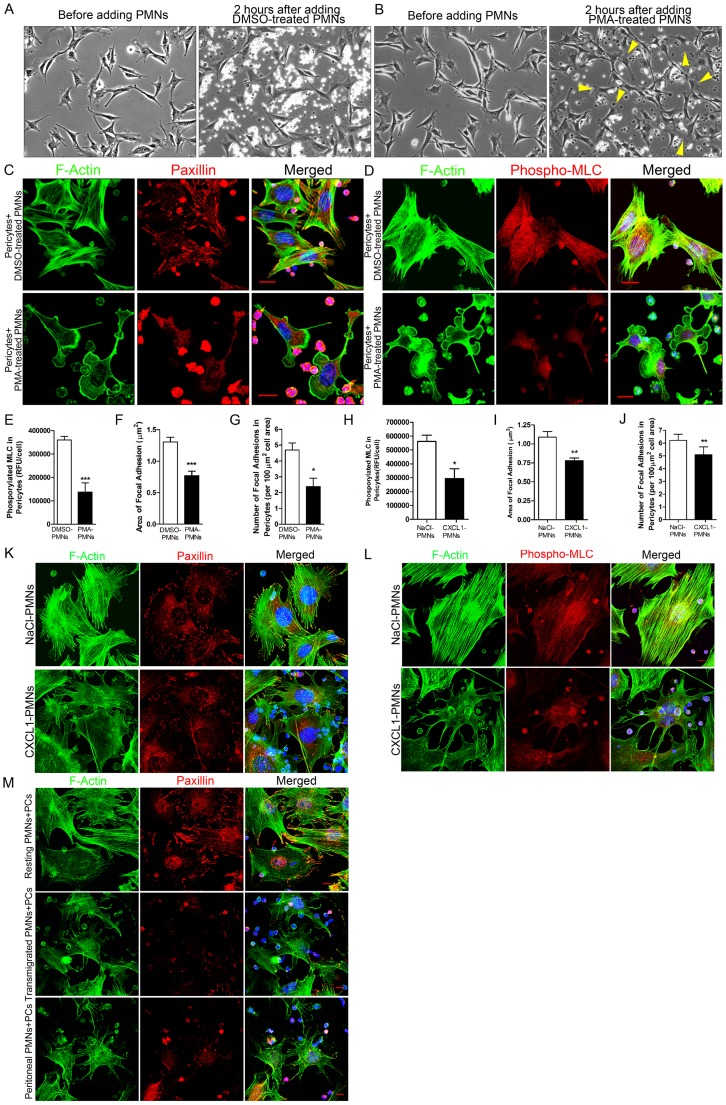
Engagement of activated PMNs induces pericyte relaxation. (**A**) Changes in mouse pericyte morphology before and after adding DMSO-treated PMNs or (**B**) before and after adding PMA-treated PMNs. (**C**) After incubation of DMSO- or PMA-treated PMNs with mouse primary pericytes, cells were fixed and stained for F-actin (green), nuclei (blue), and paxillin (red) or phosphorylated MLC (red) (**D**). (**E**) Change in the level of phosphorylated myosin light chain (phospho-MLC) in pericytes responding to DMSO- or PMA-treated PMNs. Change in the area (**F**) and the number (**G**) of focal adhesions in pericytes responding to DMSO- or PMA-treated PMNs. (**H**) Change in the level of phospho-MLC in pericytes responding to NaCl- or CXCL1-treated PMNs. Change in the area (**I**) and the number (**J**) of focal adhesions in pericytes responding to NaCl- or CXCL1-treated PMNs. T test, *P<0.05, **P<0.01 and ***P<0.001. (**K**) Response of mouse pericytes to PMNs activated by CXCL1. Cells were stained for F-actin (green), paxillin (red) and nuclei (blue). (**L**) Response of mouse pericyte to PMNs activated by CXCL1. Cells were stained for F-actin (green), phospho-MLC (red) and nuclei (blue). (**M**) Response of mouse pericytes to PMNs that have passed across an endothelial layer *in vitro* in response to IL-8 or that have been collected from the mouse peritoneal cavity following thioglycollate intra-peritoneal injection. Cells were stained for F-actin (green), paxillin (red) and nuclei (blue). Bar = 10 µm.

To examine the signaling initiated in pericytes by PMNs, we incubated mouse primary pericytes with PMNs which were pre-treated with DMSO- or phorbol 12-myristate 13-acetate (PMA), an elevator of integrin expression as well as an activator of integrins on the PMN surface [25], [26]. Analyzed by time-lapse images, no significant change in pericyte shape was observed after pericytes were stimulated by DMSO-treated PMNs (**Figure 4A**), whereas exposure to PMA-activated PMNs induced pericytes to extend prominent lamellipodia, elongate and increase their motility (**Figure 4B** **, Movie S1** and **Movie S2**). In addition, pericytes disassembled stress fibers and focal adhesions and decreased myosin light chain (MLC) phosphorylation upon stimulation of PMA-activated PMNs, indicative of cell relaxation [27] (**Figure 4C–4G** **)**.

At concentrations above 1 ng/ml, PMA induces a similar phenotype in pericytes to that caused by PMA-activated PMNs (data not shown). We were concerned that the effects of activated PMNs on pericytes might be due to PMA leaching out of the PMNs. To exclude this possibility, we developed two experimental strategies. In one of these, we cultured pericytes on two sides of a FN-coated coverslip and stimulated them with serum-free medium containing TNF-α. After washes, PMA-activated PMNs were added onto the top side of pericyte layers. Cells were fixed and stained for F-actin. We found that pericytes on the top surface (right image in **Figure S3A**) exhibited large lamellipodia and lost actin stress fibers, compared to the cells growing on the bottom surface (left image in **Figure S3A**) where contact with PMNs was not possible. In another strategy, we collected the medium from the tissue culture wells in which mouse primary pericytes had interacted with PMA- or DMSO-treated PMNs and used these conditioned medium to stimulate another batch of pericyte layers growing on FN-coated coverslips. Consistent with the aforementioned results, exposure to PMA-treated (top left image in **Figure S3B**) rather than DMSO-treated PMNs (top right image in **Figure S3B**) led to loss of actin stress fibers and focal adhesions in pericytes. However, conditioned medium collected from the wells, in which either PMA-treated or DMSO-treated PMNs had interacted with pericytes, had no influence on the morphology and cytoskeleton in the second batch of pericytes (bottom images in **Figure S3B**). These observations suggest that the pericyte response was not induced by residual PMA, but rather resulted from direct contact with activated PMNs that induced relaxation of the pericyte actomyosin system.

We also examined pericyte morphology and cytoskeletal organization in response to PMNs activated by more physiological agents. PMNs treated with murine CXCL1 (KC), an inflammatory chemokine that has neutrophil chemoattractant activity [28], induced a loss of stress fibers and focal adhesions, and decreased MLC phosphorylation (**Figure 4H–4L**). Pericytes also exhibited a relaxation phenotype (decreased stress fibers and focal adhesions) after engagement with PMNs that had been stimulated with IL-8 and collected after passage across an endothelial cell layer *in vitro* (**Figure 4M** **, middle panel**). We also induced an inflammatory response in mouse peritoneal cavities by injection of thioglycollate. PMNs were then isolated from the peritoneum and incubated with pericytes. Again, we observed that these physiologically activated PMNs induced a loss of pericyte stress fibers and focal adhesions (**Figure 4M** **, lower panel**). Additionally, a similar phenotype was seen when pericytes were exposed to PMNs that had been activated by MnCl_2_, which increases integrin affinity [29], [30] (**Figure S4**).

### Pericyte Relaxation Results from Inhibition of the RhoA/Rho Kinase (ROCK) Signaling Pathway and Suppression of Actomyosin-based Contractility

**Figure 5:**
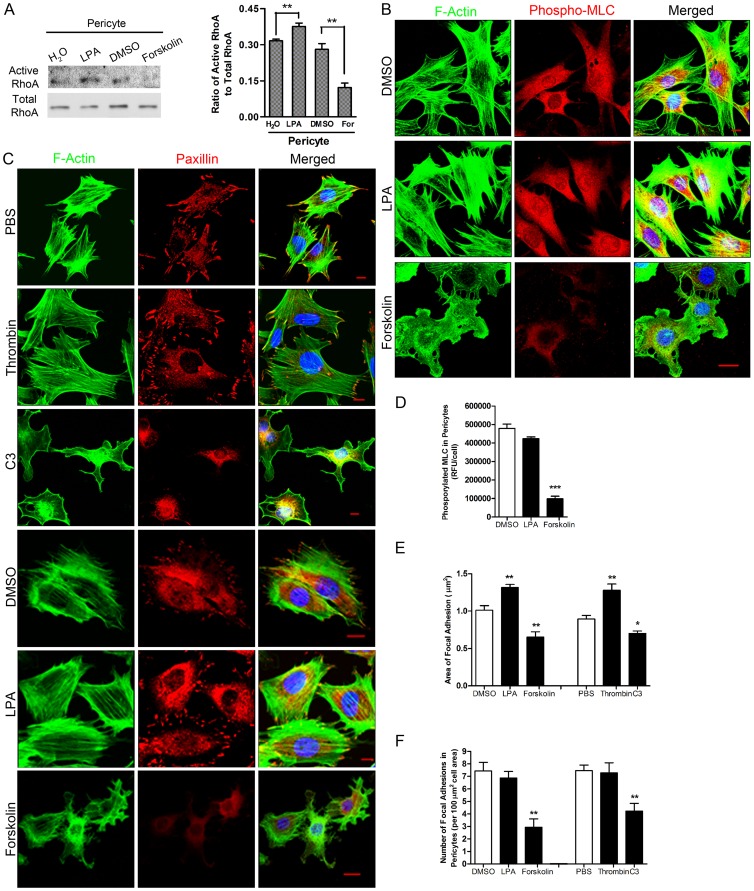
Inhibition of endogenous RhoA leads to a relaxation phenotype in pericytes. (**A**) Mouse primary pericytes were treated with LPA, Forskolin (For) or their vehicles. RhoA activity in cell lysates was detected using a pull-down assay. n = 5, t test and **P<0.01 as indicated. (**B**) Mouse primary pericytes were untreated or treated with LPA or Forskolin. Cells were fixed and stained for F-actin, phospho-MLC and nuclei (shown in blue). (**C**) Mouse primary pericytes were treated with different compounds such as Thrombin, C3, LPA and Forskolin. Cells were fixed and stained for F-actin, paxillin and nuclei (shown in blue). Bar = 10 µm. (**D**) Change in the level of phosphorylated MLC in pericytes responding to different compounds. Change in the area (**E**) and the number (**F**) of focal adhesions in pericytes responding to the different compounds. Three independent experiments and ≥10 fields/experiment were analyzed in **D, E** and **F**. T test, *P<0.05, **P<0.01 and ***P<0.001, compared to vehicles (i.e. DMSO or PBS).

Because PMN interaction with pericytes induced stress fiber disassembly, we examined the role of RhoA and its effector, ROCK [31] in regulating pericyte contractility. Pericytes in culture usually display prominent stress fibers and focal adhesions, indicative of a high level of contractility. We examined whether RhoA in cultured pericytes can be activated or inactivated after exposure to different stimuli. To this end, a series of compounds was added to primary pericyte cultures individually. Lysophosphatidic acid (LPA) [32] increased RhoA activation in mouse primary pericytes, while Forskolin, an elevator of cAMP [33], inhibited its activation (**Figure 5A**). This suggested that RhoA activation in pericytes could be either increased or suppressed in our culture conditions, depending on the type of stimulus. Morphological analysis demonstrated that RhoA activators, including LPA and thrombin [34], did not have much effect on pericyte shape. However, RhoA inhibitors such as Forskolin and C3 Transferase (C3) [35] decreased MLC phosphorylation in pericytes (**Figure 5B, 5D**) and induced loss of stress fibers and focal adhesions (**Figure 5C–5F**) resulting in a migratory phenotype. These responses are similar to those triggered by interaction with activated PMNs.

**Figure 6:**
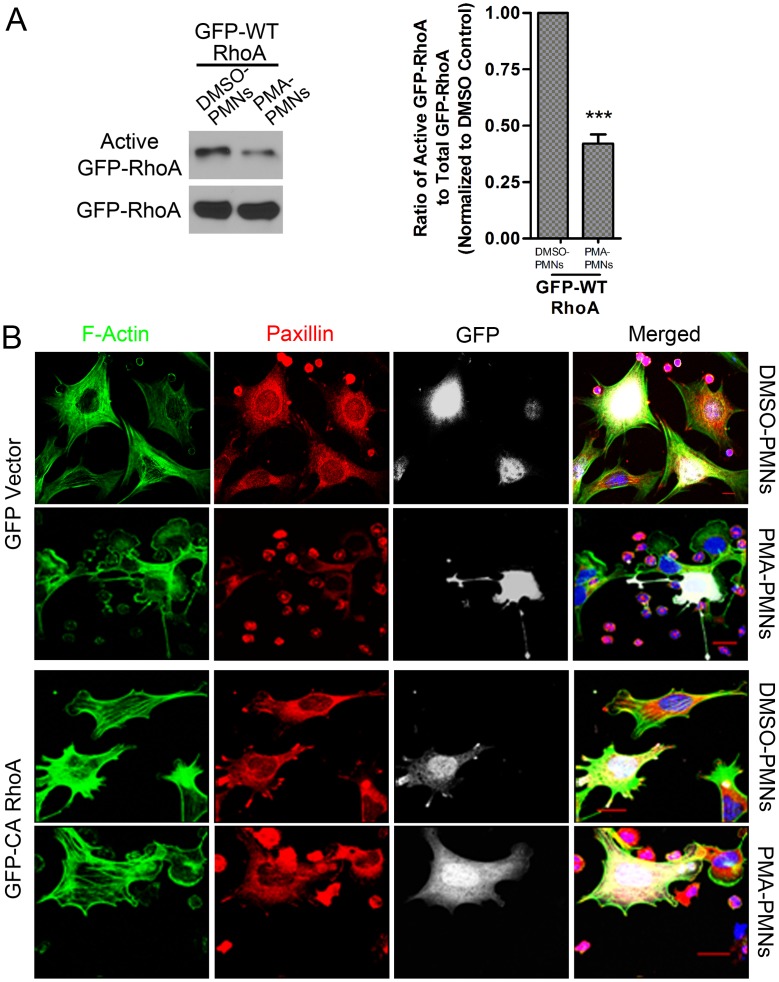
Engagement of activated PMNs inhibits RhoA activity in pericytes. (**A**) Pericytes expressing GFP-tagged WT human RhoA were exposed to DMSO-treated or PMA-treated PMNs. Activity of GFP-tagged RhoA was detected. n = 3, t test. ***P<0.001. (**B**) Pericytes expressing GFP-vector or GFP-tagged CA human RhoA were exposed to DMSO-treated or PMA-treated PMNs for one hour. Cells were fixed and stained for F-actin (green), paxillin (red), and nuclei (blue). GFP was shown in white. Expression of CA RhoA in pericytes blocked the response induced by PMA-treated PMNs. Bar = 10 µm.

In order to examine the effect of activated PMNs on RhoA activity within pericytes, we used viral vectors to express GFP-tagged WT RhoA in pericytes (**Figure S5**). In addition, we also expressed GFP-tagged constitutively active (CA) Q63L or dominant negative (DN) T19N RhoA or GFP alone in pericytes (**Figure S5**). This allowed us to distinguish RhoA in pericytes from RhoA in PMNs when investigating the two cell types together. Activated PMNs decreased the activity of exogenously expressed WT RhoA in pericytes when compared with non-activated PMNs (**Figure 6A**). However, no depression was seen in cells expressing the constitutively active RhoA (**Figure S6**). As expected, LPA activated WT RhoA, whereas Forskolin inhibited it. Neither treatment influenced the activity of CA or DN RhoA (**Figure S6**).

**Figure 7:**
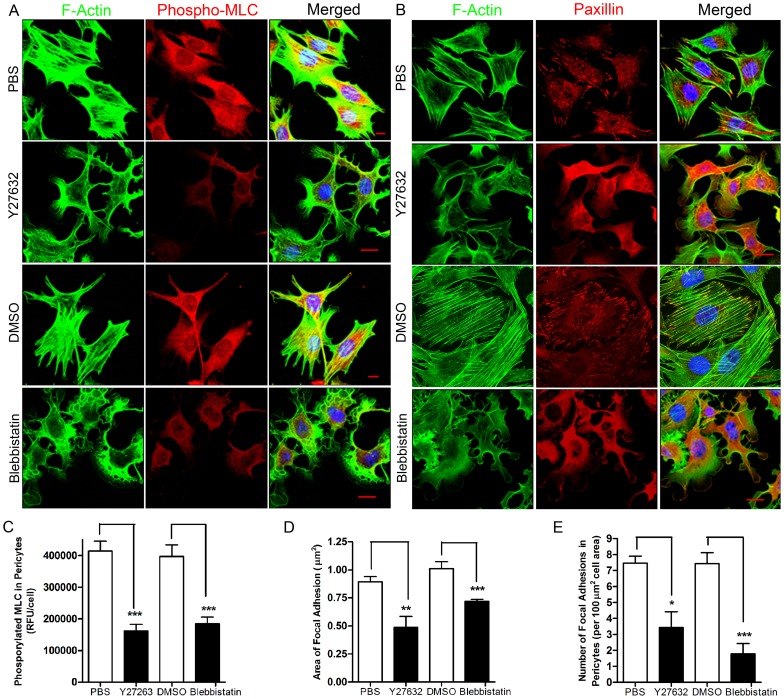
Inhibition of ROCK or myosin ATPase, downstream of RhoA, induces a relaxation phenotype in pericytes. (**A**) Mouse primary pericytes were treated with the inhibitors Y27632 or Blebbistatin. Cells were fixed and stained for F-actin, phospho-MLC and nuclei. (**B**) Mouse primary pericytes were treated with the inhibitors Y27632 or Blebbistatin. Cells were fixed and stained for F-actin, paxillin and nuclei. Bar = 10 µm. (**C**) Change in the level of phosphorylated MLC in pericytes responding to different compounds. Change in the area (**D**) and the number (**E**) of focal adhesions in pericytes responding to the different compounds. Three independent experiments and ≥10 fields/experiment were analyzed in **C, D** and **E**. T test, *P<0.05, **P<0.01 and ***P<0.001.

Compared to cells expressing GFP alone, pericytes expressing CA RhoA maintained prominent focal adhesions and stress fibers after exposure to PMA-activated PMNs (**Figure 6B**). In addition, knocking down endogenous RhoA by ShRNA led to loss of stress fibers and focal adhesions in pericytes (**Figure S5A, S5B** and **Figure S7**). We also examined pericyte response to Y27632 and Blebbistatin, inhibitors for ROCK [36] and myosin ATPase [37] respectively that are downstream of RhoA. We found that both of these compounds decreased the phosphorylation of MLC and induced disassembly of stress fibers and focal adhesions in pericytes **(** **Figure 7A–7E**). Together these results support a model in which activated PMNs induce the loss of stress fibers and focal adhesions in pericytes by inhibiting the RhoA-ROCK pathway leading to reduced pericyte actomyosin-based contractility [31], [38].

### Pericyte Relaxation Affects the Morphology of Pericyte Monolayers

**Figure 8:**
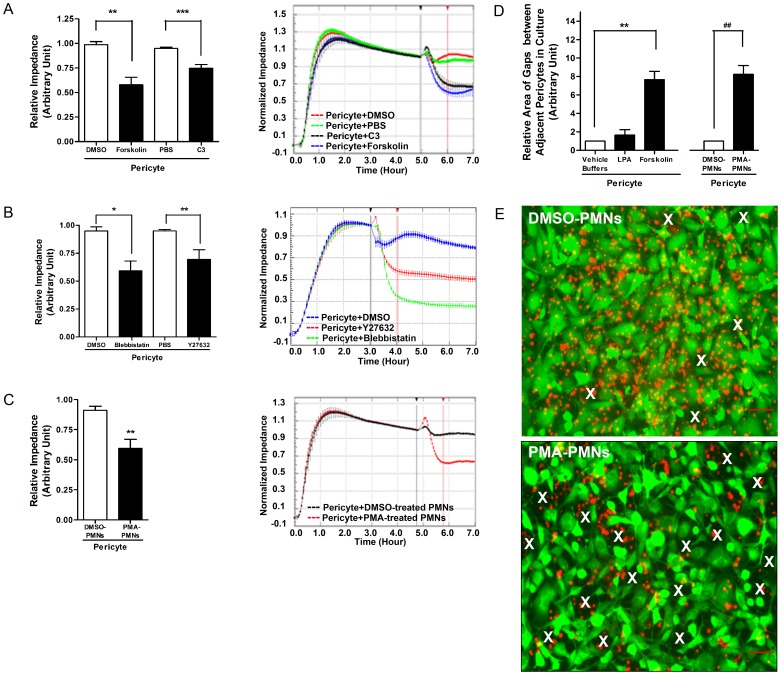
Relaxation of pericytes expands pericyte gaps in a pericyte monolayer. (**A**)–(**C**) On the left, the relative impedance is shown for pericytes responding to different treatments. Representative real-time read-outs are shown on the right. The relative impedance at 1 hour after adding a chemical (indicated by the red vertical line) was normalized to the impedance when reagents or cells were added (indicated by the black vertical line). In (**A**), the effects of Forskolin or C3 were analyzed, and in (**B**), the effects of Y27632 or Blebbistatin were compared. (**C**) the effect of DMSO- or PMA-treated PMNs on the impedance of primary pericyte monolayers. (**D**) Change in area of pericyte gaps in culture. Calcein green-labeled pericytes were grown to confluence on FN-coated coverslips in the wells of a 24 well plate. Compounds such as LPA or Forskolin, or Calcein blue-labeled mouse PMNs, pretreated with DMSO or PMA, were added to pericyte monolayers for 1 hour. Cells were fixed and imaged. The area of pericyte gaps was measured. (**E**) Representative images showing the change in size of gaps between pericytes upon stimulation of DMSO- or PMA-pretreated PMNs. Prominent pericyte gaps are indicated by white X’s. Note: The same amount of DMSO- or PMA-treated PMNs were added onto pericyte monolayers. However, due to the change in pericyte morphology and PMA-treated PMN transmigration, the density of both PMNs and pericytes looks lower in the PMA-treated group. Bar = 50 µm. n = 4–5. T test as indicated. *P<0.05, ** or ^##^P<0.01, ***P<0.001.

Our results suggest that the pericyte sheath serves as a barrier to leukocyte extravasation. We wished to examine the barrier properties of pericytes further and particularly the formation of gaps between pericytes in tissue culture. However, unlike endothelial cells, pericytes do not form monolayers in which adjacent cells are held together by adherent junctions and tight junctions. Consequently, conventional electrical resistance measurements are not a practical approach for monitoring changes in permeability as PMNs migrate across the pericyte monolayer. However, electrical impedance measurements provide a measure of cell area and adhesion to the underlying substratum [39], [40] and we have taken advantage of this approach. Compounds decreasing actomyosin contractility, including Forskolin, C3, Y27632 and Blebbistatin, decreased the impedance of pericyte layers and led to a relaxation phenotype of pericytes (**Figure 8A, 8B** **,** **Figure 5** and **Figure7**). However, agents that increased RhoA activity slightly increased or had no effect on impedance (data not shown). Incubating the pericytes with PMA-activated or MnCl_2_-activated PMNs reduced impedance of the pericyte layer (**Figure 8C** and **Figure S4**). Examining pericyte monolayers revealed that the spaces between pericytes were enlarged by activated PMNs and by the presence of relaxants, such as Forskolin or C3 (**Figure 8D, 8E** **)**. Agents that stimulate contraction, such as LPA, did not induce or expand the gaps between pericytes (**Figure 8D**).

### Pericyte Relaxation Facilitates PMN Transmigration both *in vitro* and *in vivo*

**Figure 9:**
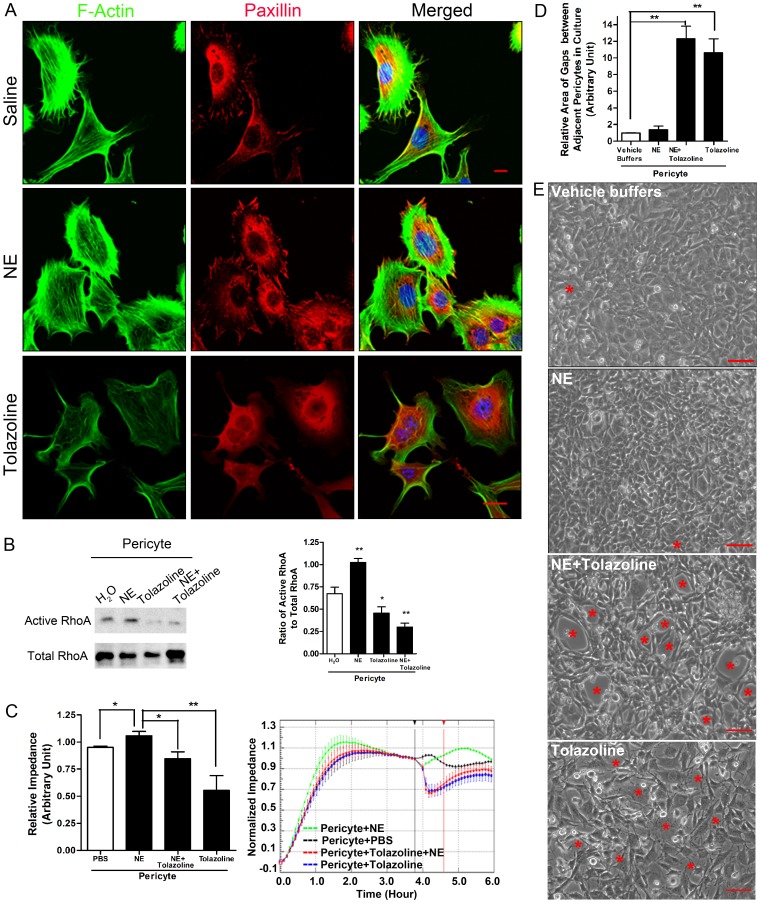
Effect of vasoactive drugs, NE and Tolazoline, on pericyte morphology and permeability of pericyte monolayers. (**A**) Mouse primary pericytes were treated with buffer, NE or Tolazoline. Cells were fixed and stained for F-actin, paxillin and nuclei. Bar = 10 µm. (**B**) NE increased RhoA activity in pericytes while Tolazoline inhibited it. NE or Tolazoline were added to pericytes and RhoA activity was measured in a pull-down assay. n = 5. T test. *P<0.05 and **P<0.01, compared to vehicle. (**C**) Effects of NE, Tolazoline or NE + Tolazoline on the impedance of a pericyte monolayer were compared. On the left, the relative impedance was shown for pericytes responding to different treatments. Representative real-time read-outs were shown on the right. The relative impedance at 1 hour after adding a chemical (indicated by the red vertical line) was normalized to the impedance when reagents or cells were added (indicated by the black vertical line). n = 4–5. T test as indicated. P<0.05 and **P<0.01. (**D**) Change in area of pericyte gaps in culture. Pericytes were grown to confluence on FN-coated coverslips in the wells of a 24 well plate. NE, tolazoline, or NE plus Tolazoline were added to pericyte monolayers for 1 hour. Cells were fixed and imaged. The area of pericyte gaps was measured. n = 5. T test as indicated. **P<0.01. (**E**) Representative images showing the change in size of pericyte gaps upon stimulation of vehicle buffer, NE or NE plus Tolazoline. Pericyte gaps are indicated by red *’s. Bar = 50 µm.

Our data are consistent with a model in which PMN interaction with pericytes induces pericyte relaxation and a consequent expansion of the gaps between pericytes, thereby facilitating passage of PMNs across the pericyte sheath. We wished to determine whether agents that affect pericyte contractility in vivo may affect PMN passage across the pericyte layer. Two drugs that have been used clinically to induce vasoconstriction or vasodilation are Norepinephrine (NE) and Tolazoline, respectively. NE is an agonist [41] of α-adrenergic receptors in pericytes and smooth muscle cells, whereas Tolazoline is an antagonist [42]. We first examined the response of pericytes to these agents in pericyte cultures. Our data demonstrated that pericytes maintained their actin stress fibers and focal adhesions in response to NE but lost these structures in the presence of Tolazoline (**Figure 9A**). NE activated RhoA and slightly increased the impedance of pericyte monolayers, whereas Tolazoline decreased RhoA activity and decreased the impedance of cultures (**Figure 9B, 9C** **)**. Image analysis of pericyte monolayers revealed that gaps between pericytes were enlarged by the presence of Tolazoline whereas NE did not induce or expand the gaps between pericytes (**Figure 9D, 9E** **)**.

**Figure 10:**
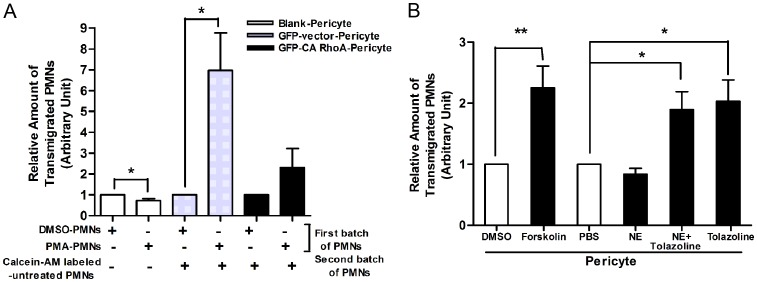
Relaxation of pericytes facilitates PMN transmigration *in vitro*. (**A**) Pericyte engagement with PMA-activated PMNs enhanced PMN passage across a pericyte monolayer. n = 5. Note: Due to strong adhesion to the pericytes and the filters, migration of PMA-activated PMNs toward IL-8 in the lower chamber was less than that of control PMNs (See the first two bars). (**B**) PMN transmigration through a pericyte monolayer in the presence of agents that affect pericyte contractility. n = 4. *P<0.05 and **P<0.01 as indicated.

We next examined the ability of PMNs to pass across pericyte monolayers grown on FN-coated filters of a Boyden transwell system. Due to strong adhesion to the pericytes and the filters, migration of PMA-activated PMNs toward IL-8 in the lower chamber was less than control PMNs (See the first two bars in **Figure 10A**). However, as discussed above, engagement of activated PMNs expands pericyte gaps. Pre-exposure of pericyte monolayers to activated PMNs induced an increased transmigration (six fold) of a second batch of Calcein-AM-labeled resting PMNs. This response was diminished when the pericyte layers expressed CA RhoA (**Figure 10A**). In addition, more PMNs passed through pericyte layers pretreated with relaxants such as Forskolin and Tolazoline than through layers treated with NE or vehicles (e.g. DMSO or PBS) (**Figure 10B**). These findings indicate that migration of PMNs across pericyte monolayers is promoted by inhibiting pericyte contractility, which results in the expansion of gaps between pericytes.

**Figure 11:**
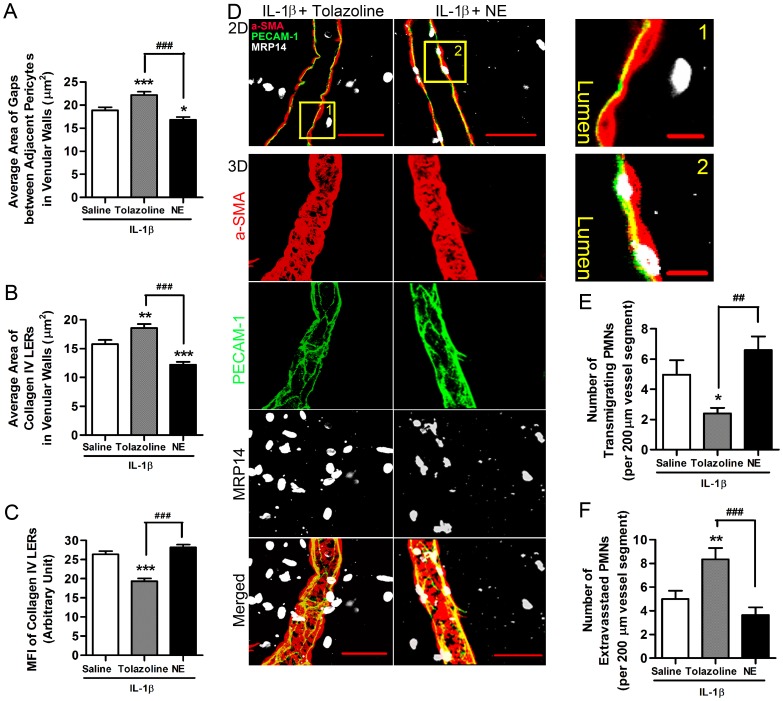
Relaxation of pericytes facilitates PMN transmigration *in vivo*. (**A**) Change in area of pericyte gaps in IL-1β-stimulated venular walls exposed to Tolazoline or NE. (**B**) Change in area of collagen IV LERs in IL-1β-stimulated venules treated with Tolazoline or NE. (**C**) Change in MFI of collagen IV LERs in IL-1β-stimulated venular walls treated with Tolazoline or NE. (**D**) Representative images of IL-1β-stimulated cremaster muscle venules illustrating the effect of Tolazoline and NE on PMN transmigration *in vivo*. The top panels show 2D images from the middle sections of these venules, stained as indicated to reveal transmigrating and extravasated PMNs. Higher magnifications of the boxed areas (marked with Arabic numerals) are displayed on the right. The bottom 3D images are reconstructions of the same venules stained as indicated to reveal extravasated PMNs. (**E**) Number of PMNs in the process of transmigrating in IL-1β-stimulated cremasters treated with saline, Tolazoline or NE. (**F**) Number of extravasated PMNs in the perivascular tissues 100 µm around a 200 µm vessel segment in IL-1β-injected mouse cremaster muscles. 7–8 mice and 15 vessel segments from each mouse were analyzed in each group of the experiments displayed. Bar = 10 µm in the zoom-in views, and = 50 µm in other images. ANOVA plus multiple comparisons were performed. Comparisons with saline control were displayed by *P<0.05, **P<0.01, and ***P<0.001. Other comparisons were indicated as ^##^P<0.01 and ^###^P<0.001.

To examine whether modulating the contractility of pericytes affects PMN transmigration *in vivo*, we topically applied NE or Tolazoline to exteriorized cremaster muscles which had been pre-stimulated with IL-1β. Treatment with Tolazoline significantly enlarged pericyte gaps (**Figure 11A**). In addition, Tolazoline induced an expansion of the LERs and caused these to become thinner (i.e. there was decreased collagen fluorescence intensity) (**Figure 11B, 11C**). Examining the distribution of PMNs, we observed that Tolazoline released PMNs from the space between ECs and pericytes, reducing the number of PMNs within the vessel wall (**Figure 11D, 11E**) but increasing the number of extravasated leukocytes (**Figure 11D, 11F**). In contrast, NE decreased the area of pericyte gaps and LERs, compared to a saline control (**Figure 11A, 11B**). NE tended to increase the number of PMNs in the space between ECs and pericytes but decrease the number of PMNs that successfully completed extravasation (**Figure 11D–11F**). These *in vivo* data further support that relaxation of pericytes expands the gaps between them, as well as the adjacent LERs, to facilitate PMN passage across inflamed venular walls.

## Discussion

The passage of leukocytes across the venule wall is a critical event during inflammation that allows the recruitment of leukocytes out of the blood circulation to sites of infection or tissue damage. In venular walls the endothelium forms the first barrier to transmigrating leukocytes while the pericyte sheath, which is embedded in the vascular BM, provides a second barrier surrounding the endothelium [4]. Most attention has been paid to how leukocytes interact with and cross the endothelium, whereas little work has been directed toward understanding the passage of leukocytes across this second barrier [4], [5], [6]. However, migrating leukocytes tend to accumulate in the space between the endothelium and the vascular BM [7], [8] indicating the potential importance of this barrier. Previous studies demonstrated that PMNs crossing the venular BM selectively targeted regions of low extracellular matrix expression, which have become known as LERs, and that these sites tended to align with gaps between pericytes [9]. In addition, these previous studies discovered that LERs were plastic with respect to their size and thickness and underwent remodeling during leukocyte transmigration, creating channels for leukocyte extravasation [9], [10]. Although enzymatic cleavage of matrix proteins in the vascular BM has been suggested as a potential mechanism underlying LER remodeling at sites of acute inflammation [9], [43], monocytes seem not to rely on proteolysis of the vascular BM during their extravasation [43], implying that other factors may be involved in inflammation-induced LER remodeling. In this earlier work, pericytes were simply treated as reference points to indicate the location of LERs in the vascular BM. The potential role of pericytes in regulating LER remodeling and hence leukocyte extravasation was not addressed. However, the location of pericytes and their morphology (i.e. forming a sheath that is embedded in the vascular BM surrounding the vessel walls with gaps between adjacent pericytes aligning with LERs), coupled with their capacity to contract or relax [11], imply that structural changes in the pericyte sheath may influence PMN transmigration. Here we have investigated leukocyte interactions with pericytes and discovered that engagement with PMNs induces expansion of pericyte gaps and thinning of their associated LERs. Working with pericytes in culture, we show that activated PMNs bind to pericytes and initiate a signaling cascade that inhibits the RhoA/ROCK pathway, thereby inducing pericyte relaxation.

Our current data reveal pericytes to be active participants in the remodeling of LERs during PMN transmigration. This is distinct from the pericyte-mediated BM remodeling that occurs in some patho-physiological conditions such as angiogenesis [44]. In the latter case, BM remodeling is a long-term event that involves both degradation and reassembly of matrix proteins [45] and even the renewal of pericytes.

To get at the role of PMN interactions with pericytes in the regulation of LERs and pericyte gaps, we used a number of strategies to perturb PMN transit across venule walls during inflammation. Using IL-1β to induce local inflammation in mouse cremaster muscles, we were able to quantify the size of LERs, pericyte gaps and their relationship to migrating PMNs. Treatment with IL-1β induced expansion of pericyte gaps and LERs. We noted that PMN extravasation usually occurs preferentially in some venule segments but not others that are nearby and appear similar. The reason for these “hot spots” of PMN migration out of inflamed venules is not clear and merits further investigation. However, within the venular regions of preferred PMN transit, we noted that the pericyte gaps and LERs associated with transmigrating leukocytes were bigger than those in other vessel segments suggesting that the presence of leukocytes contributed to the expansion of these regions. To explore this further we depleted circulating leukocytes and this decreased both the size of the pericyte gaps and the LERs within the BM, as judged by collagen IV staining. This effect could be due to soluble factors secreted by the PMNs or the result of direct PMN-pericyte contacts. To determine whether cell-cell contact might be involved in this effect of PMNs on pericytes, we took advantage of previous studies indicating that transmigrating PECAM-1^−/−^ PMNs frequently become trapped in the IL-1β-stimulated vascular BM at a relatively early stage of acute inflammation before they reach the pericyte sheath. These cells fail to penetrate the vascular BM due to their inability to express α_6_ integrin [17], [20], which is required for migrating PMNs to bind to the vascular BM. Using IL-1β-injected PECAM-1^−/−^ mice as tools, we found separation of PMNs from the pericyte sheath by the vascular BM in these mice. Notably, we observed a decreased expansion of pericyte gaps and LERs compared with wild type mice. We also used anti-ICAM-1 and anti-α_6_ integrin antibodies to block transmigration at the stages before PMNs meet the pericyte sheath. Perfusing animals with these antibodies led to smaller size of pericyte gaps and LERs. Together these observations suggest that direct contacts between migrating PMNs and pericytes contribute to the opening of pericyte gaps and the expansion of LERs.

To explore the signaling pathway triggered by PMN/pericyte interactions, we used PMNs activated in several ways. For many experiments, we have used PMA to induce activation, but to obtain more physiologically relevant activated PMNs, we have activated some with CXCL1 or we have used PMNs retrieved after they have been stimulated by IL-8 to pass through an endothelial monolayer. We also isolated PMNs from the peritoneal cavity after induction of inflammation by intra-peritoneal injection of thioglycollate. In a few experiments, PMN integrins were selectively activated by exposure to MnCl_2_. We found that activated PMNs bound to pericytes, induced a change in pericyte shape, increased the size of gaps between pericytes in culture and promoted PMN passage across this cell layer. We were surprised to observe that in response to the activated PMNs, the pericytes disassembled their stress fibers and focal adhesions and decreased MLC phosphorylation, indicative of decreased pericyte contractility. Only when pericytes directly engaged with activated PMNs, did these reactions occur. In contrast, pericytes maintained their morphology and sub-cellular structures such as stress fibers and focal adhesions when pericytes were stimulated by the conditioned medium collected from the tissue culture wells in which activated PMNs had interacted with another batch of pericytes. These observations suggested that direct engagement with PMNs was essential to induce this response. The loss of stress fibers and focal adhesions in pericytes responding to activated PMNs suggested that this may involve inhibition of the RhoA/ROCK pathway in pericytes [31], [46]. Consistent with this possibility, expression of constitutively active RhoA in pericytes blocked this response. Inhibiting RhoA, ROCK or myosin ATPase in control cultures of pericytes induced a phenotype similar to that induced by activated PMNs. Because of the difficulty of measuring endogenous pericyte RhoA activity in mixed cultures containing both PMNs and pericytes, we expressed epitope-tagged versions of RhoA in pericytes. Activated PMNs decreased the activity of RhoA in the pericytes. This was not seen when the pericytes were incubated with PMNs that had not been activated. These results suggested that engagement of activated PMNs with mouse primary pericytes enlarges pericyte gaps by inducing pericyte relaxation via inhibition of the RhoA/ROCK pathway and suppression of actomyosin-based contractility [46]. Future efforts will be directed at identifying the adhesion molecules on pericytes and PMNs that mediate this interaction and decrease pericyte RhoA activity.

The finding that pericyte relaxation expands the gaps between these cells is unexpected and contrasts with the opening of EC gaps during neutrophil transendothelial migration, which is associated with RhoA activation and increased tension on EC junctions [47], [48]. How might relaxation of pericytes expand the gaps between them? Neutrophil engagement with pericytes in culture induces the pericytes to become more elongated and narrow, in association with increased motility. Increased pericyte motility has been observed in some acute conditions such as ischemia and reperfusion *in vivo* [49], and may result in long term remodeling of the matrix. While cell shape changes may contribute to the expansion of pericyte gaps, pericyte relaxation leading to vasodilation may also be a factor. Pericyte contractility regulates the diameter of capillaries [50] and the contractile properties of pericytes may similarly regulate venule diameter. Indeed, venule dilation has been observed during the late stages of inflammation and is accompanied by leukocyte margination [51]. Venule dilation should stretch the pericyte sheath, expanding the gaps between pericytes because the adhesions between pericytes appear to be much weaker than their adhesions to the BM. Stretching the vascular BM between pericytes should also contribute to thinning of the LERs. It is interesting that ECs and pericytes contribute to the regulation of neutrophil extravasation by seemingly opposite mechanisms.

We argued that if our model is correct and the size of the pericyte gaps and the LERs is governed by the contractile state of the pericytes, we ought to be able to manipulate the contractile state of pericytes directly and thereby affect gap size and influence PMN migration through inflamed venule walls. To enhance pericyte contraction we applied NE and to relax pericytes we applied Tolazoline locally to IL-1β-treated cremaster muscles. Whereas NE slightly decreased the size of the pericyte gaps or LERs and tended to increase the number of PMNs in the space between ECs and pericytes, Tolazoline induced a significant expansion of the gaps and LERs. Additionally, Tolazoline reduced the number of PMNs within the venule wall and increased the number of PMNs that successfully extravasated through the venule wall into the adjacent tissue. Together our results indicate that the transit of leukocytes across inflamed venule walls is regulated in part by the contractile state of pericytes that controls the size of the LERs in the venular BM and the dimensions of the gaps between pericytes. Previous work has described LERs as “gates” for leukocyte transit through the venular wall [4], [9], [10] but has not addressed the mechanisms controlling the opening of these gates. Our work suggests that pericytes play a key role in regulating the size of these gates through which leukocytes migrate, following a potential pathway that is illustrated in **Figure S8**. As such, pericytes present a potential target for the development of therapeutic agents aimed at decreasing leukocyte recruitment in inflammatory diseases.

Compared to the extensive literature examining how PMNs interact with endothelial cells during PMN extravasation, our understanding of the role of pericytes in this event is just starting. In addition, some physical and functional associations exist between endothelial cells and pericytes in venular walls and during the development of the vasculature [11], [52], [53]. The role of these interactions, which has generally been ignored, will need to be investigated during PMN diapedesis.

After this work was completed, a paper by Proebstl et al. was published analyzing the migration of neutrophils across venular walls during inflammation [54]. In that study, neutrophils were imaged crossing the endothelium and then migrating beneath the endothelial cells and along pericyte processes. The neutrophils were observed to pass through gaps between the pericytes, as we have described here. Like our work, they showed that inflammation induced expansion of the gaps. However, in this other study, the expansion of the gaps between pericytes was attributed solely to the effects of inflammatory cytokines, whereas we provide evidence that, in addition to the action of inflammatory cytokines, the interaction of leukocytes with pericytes contributes to the enlargement of the pericyte gaps. In the present study we also provide evidence that the expansion of the gaps is due to a decrease in the RhoA/ROCK signaling pathway that results in relaxation of the pericytes.

## Supporting Information

Figure S1 **Expression of α-SMA in microvasculature and occurrence of PMN extravasation. (A)** Representative images show different types of vessel segments, among which PMN extravasation occurred primarily at the post-capillary venule. Mouse cremaster muscles were injected with IL-1β. Two hours later, tissues were collected, immuno-stained for α-SMA, MRP14 and PECAM-1, and imaged in 3D. The areas outlined in the merged image panel are shown magnified in the corresponding panels as indicated, so as to reveal the expression of α-SMA and PECAM-1 in the arteriole, capillary and venule. Note: due to the relatively higher expression of α-SMA in the arteriole, the arteriole image intensity was saturated. Bar = 10 µm. **(B)** Quantitation of PMNs extravasated from capillaries, post-capillary venules or arterioles in mouse cremaster muscles or skin which were stimulated by either IL-1α or TNF-α. Ten vessel segments/mouse and four mice/group were analyzed. ANOVA plus Neuman-Keuls multiple comparisons were performed as indicated. *** P<0.001. **(C)** TNF-α-injected mouse cremaster muscles were immuno-stained for smooth muscle actin, PECAM-1 and MRP14 and imaged in 3D. α-SMA staining in capillaries was faint and even negative (indicated by yellow arrows in the merged image) but strong in post-capillary venules (indicated by a white solid arrow). PMN extravasation took place at the post-capillary venule. Bar = 10 µm.
(TIF)

Figure S2 **α-SMA staining in an arteriole, a post-capillary venule, and a collecting venule of mouse cremaster muscles.** Resting tissues were immuno-stained for α-SMA, imaged in 3D and displayed at high magnification. In the arteriole, α-SMA positive cells (i.e. smooth muscle cells) regularly surrounded the vessel like compact coils. However, α-SMA positive cells intricately presented in the post-capillary venule and extended a number of longitudinal and circumferential processes (indicated by green arc arrows), exhibiting a typical pericyte phenotype. Gaps between adjacent cells were marked by yellow arrows. In the collecting venule, α-SMA positive cells (i.e. muscle cells) regularly coiled around the vessel wall.
(TIF)

Figure S3 **Direct contacts with PMA-activated PMNs lead to pericyte relaxation.** To further confirm that pericyte responses to PMA-treated PMNs were mediated by direct cell-cell contacts between PMNs and pericytes rather than by the residual PMA in the culture medium, we developed two experimental strategies as described below. **(A)** In serum-free medium containing TNF-α (15 ng/ml), mouse primary pericytes were grown on two sides of a FN-coated coverslip placed in the well of a 24 well plate overnight. After being extensively washed, PMA-treated PMNs were added on the pericyte layer growing on the top surface of coverslips for one hour. Cells were fixed and stained for F-actin and nuclei. Pericytes on the top surface (left image) exhibited large lamellipodia and lost actin stress fibers, compared to the cells growing on the bottom surface (right image). **(B)** In serum-free medium containing TNF-α (15 ng/ml), mouse primary pericytes were grown on FN-coated coverslips placed in the well of a 24 well plate overnight. After being extensively washed, PMA- or DMSO-treated PMNs were added. One hour later, the conditioned medium was collected from these wells and any contaminating cells (probably including some pericytes and PMNs) were removed by centrifugation. These conditioned media were added to another batch of pericytes growing on FN-coated coverslips. Cells were fixed and stained for F-actin, paxillin and nuclei. Exposure to PMA-treated (top left image) rather than DMSO-treated PMNs (top right image) led to loss of actin stress fibers and focal adhesions in pericytes. However, conditioned medium collected from the wells, in which either PMA-treated (bottom left image) or DMSO-treated PMNs (bottom right image) had interacted with pericytes, had no influence on the morphology and cytoskeleton in the second batch of pericytes. Arrowheads indicate PMNs. Bar = 10 µm. Data shown in **(A)** and **(B)** suggest that direct contacts with PMA-activated PMNs mediate pericyte relaxation.
(TIF)

Figure S4 **Engagement of MnCl_2_-activated PMNs leads to pericyte relaxation and increases permeability of pericyte monolayers. (A)** TNF-α-induced pericytes were grown on FN-coated coverslips. NaCl- or MnCl_2_-treated PMNs that had been extensively washed after stimulation were added for 1 hour. Cells were fixed and stained for nuclei, F-actin and paxillin. MnCl_2_-treated PMNs induced partial loss of actin stress fibers and focal adhesions in pericytes. **(B)** TNF-α-induced mouse primary pericytes were seeded on the wells of a RTCA analyzer for 3 to 5 hours to make a confluent cell monolayer. NaCl- or MnCl_2_-treated PMNs were respectively added to each well for one more hour. The impedance of a pericyte monolayer was recorded in time-lapse. As demonstrated by the representative experimental curves in the right graph, the relative impedance at 1 hour after adding NaCl- or MnCl_2_-treated PMNs (indicated by red vertical line) was normalized to that when PMNs were added (indicated by black vertical line). Exposure to MnCl_2_-treated PMNs caused a decrease of the impedance in pericyte monolayers (left bar graph). T test, *P<0.05.
(TIF)

Figure S5 **Sequence of shRNA specific to mouse RhoA and expression of exogenous GFP-targeted RhoA.** To analyze the role of RhoA in pericytes interacting with PMNs, we infected mouse primary pericytes with lentivirus bearing a GFP reporter and short-hairpin RNA (shRNA) to knock down RhoA in mouse pericytes. In some cases, we expressed exogenous GFP-tagged wild type (WT), constitutively active (CA) Q63L or dominant negative (DN) T19N RhoA in mouse primary pericytes. This allowed us to distinguish RhoA in pericytes from RhoA in PMNs when investigating pericyte interactions with PMNs. **(A)** ShRNA-targeted sequence of mouse RhoA is displayed, as well as the expression levels of endogenous RhoA in mouse MEF cells and primary pericytes as revealed by western blots in control cells or following knockdown by the shRNA. **(B)** Expression of exogenous GFP-tagged human RhoA in mouse primary pericytes was revealed by western blots. **(C)** Representative images showed expression of GFP or GFP-tagged RhoA in mouse primary pericytes. Nuclei were displayed in blue. Approximately 90% GFP positive cells were achieved. Bar = 50 µm.
(TIF)

Figure S6 **Assay of GFP-tagged RhoA in pericytes after exposure to different chemicals or engagement with PMNs.** Pericytes expressing GFP-tagged CA, DN or WT human RhoA were starved in serum-free medium supplied with TNF-α overnight and exposed to the indicated reagents or to DMSO-treated or PMA-treated PMNs. Activity of GFP-tagged RhoA was detected (n = 7 for pericytes treated with chemicals and n = 3 for pericytes interacting with PMNs. t test. * P<0.05 and ** P<0.01 as indicated).
(TIF)

Figure S7 **Knocking down RhoA expression leads to a relaxation phenotype in pericytes.** Mouse pericytes were infected with lentivirus carrying GFP empty vector, or GFP-tagged human WT RhoA or shRNA specific to mouse RhoA. Cells were seeded on FN-coated coverslips and stained for F-actin (green), paxillin (red) and nuclei (blue). Samples were imaged and GFP was displayed in white. Compared to GFP-negative cells or those expressing GFP empty vector or GFP-tagged WT RhoA, pericytes bearing shRNA for RhoA extended protrusions and lost stress fibers and focal adhesions. Bar = 10 µm.
(TIF)

Figure S8 **A diagram illustrating the interactions of PMNs migrating across a venule wall.** The top diagram demonstrates the structure of a venule wall, illustrating that the endothelium contacts the vascular basement membrane with its abluminal surface, whereas the pericyte sheath is embedded in this basement membrane. In some cases pericytes may also establish interactions with endothelial cells via extended processes (not shown). The diagram also shows PMNs at different stages of migration. The boxed areas (**A** and **B**) in the top diagram are displayed at higher magnification in the corresponding bottom panels. PMN  =  Polymorphonuclear neutrophil; EC  =  endothelial cell; BM  =  Basement membrane; PC  =  Pericyte; TEM  =  Trans-endothelial migration; TBMM  =  Trans-basement membrane migration; TPM  =  Trans-pericyte migration. Box **A** shows a transmigrating PMN that has broken through the endothelium but is separated from the pericyte sheath by the vascular BM. In this situation, pericytes still maintain most of their actin bundles and focal adhesions which support the binding of pericytes to the BM. Diffusible inflammatory mediators (e.g. nitric oxide or complement C3a/5a) may relax pericytes to a certain extent, but this is not indicated in the diagram. At the stage shown in box **B**, the transmigrating PMN has penetrated the BM and established direct contacts with the pericyte sheath. Direct engagement with the migrating PMN elicits signals to induce pericyte relaxation disassembling actin stress fibers. Relaxation of pericytes expands the gaps between them and thins (or opens) the BM at LERs which align with pericyte gaps, thereby facilitating PMN transmigration.
(TIF)

Movie S1 **Response of pericytes to DMSO-treated PMNs.** Mouse retinal primary pericytes were seeded on the FN-coated glass surface of culture dishes for 4 hours to allow cell spreading. Cells were imaged in time-lapse at 37°C for 1 hour using the setting for phase contrast and then washed DMSO-treated PMNs were added. Interaction of pericytes and PMNs were further recorded for 2 hours after adding PMNs.
(AVI)

Movie S2 **Response of pericytes to PMA-treated PMNs.** Mouse retinal primary pericytes were seeded on the FN-coated glass surface of culture dishes for 4 hours to allow cell spreading. Cells were imaged in time-lapse at 37°C for 1 hour using the setting for phase contrast and then washed PMA-treated PMNs were added. Interaction of pericytes and PMNs were further recorded for 2 hours after adding PMNs.
(AVI)

## References

[1] Pearse D, Jarnagin K (2010) Abating progressive tissue injury and preserving function after CNS trauma: The role of inflammation modulatory therapies. Curr Opin Investig Drugs 11: 1207–1210. 21189657

[2] Ambrosio G, Weisman HF, Mannisi JA, Becker LC (1989) Progressive impairment of regional myocardial perfusion after initial restoration of postischemic blood flow. Circulation 80: 1846–1861. 2598442 10.1161/01.cir.80.6.1846

[3] Smith JA (1994) Neutrophils, host defense, and inflammation: a double-edged sword. J Leukoc Biol 56: 672–686. 7996043 10.1002/jlb.56.6.672

[4] Nourshargh S, Hordijk PL, Sixt M (2010) Breaching multiple barriers: leukocyte motility through venular walls and the interstitium. Nat Rev Mol Cell Biol 11: 366–378. 20414258 10.1038/nrm2889

[5] Muller WA (2010) Mechanisms of leukocyte transendothelial migration. Annu Rev Pathol 6: 323–344. 10.1146/annurev-pathol-011110-130224 PMC3628537 21073340

[6] Rowe RG, Weiss SJ (2008) Breaching the basement membrane: who, when and how? Trends Cell Biol 18: 560–574. 18848450 10.1016/j.tcb.2008.08.007

[7] Hurley JV (1963) An electron microscopic study of leucocytic emigration and vascular permeability in rat skin. Aust J Exp Biol Med Sci 41: 171–186. 13955841 10.1038/icb.1963.17

[8] Yadav R, Larbi KY, Young RE, Nourshargh S (2003) Migration of leukocytes through the vessel wall and beyond. Thromb Haemost 90: 598–606. 14515179 10.1160/TH03-04-0220

[9] Wang S, Voisin MB, Larbi KY, Dangerfield J, Scheiermann C, et al (2006) Venular basement membranes contain specific matrix protein low expression regions that act as exit points for emigrating neutrophils. J Exp Med 203: 1519–1532. 16754715 10.1084/jem.20051210 PMC2118318

[10] Voisin MB, Probstl D, Nourshargh S (2010) Venular basement membranes ubiquitously express matrix protein low-expression regions: characterization in multiple tissues and remodeling during inflammation. Am J Pathol 176: 482–495. 20008148 10.2353/ajpath.2010.090510 PMC2797906

[11] Diaz-Flores L, Gutierrez R, Madrid JF, Varela H, Valladares F, et al (2009) Pericytes. Morphofunction, interactions and pathology in a quiescent and activated mesenchymal cell niche. Histol Histopathol 24: 909–969. 19475537 10.14670/HH-24.909

[12] Armulik A, Abramsson A, Betsholtz C (2005) Endothelial/pericyte interactions. Circ Res 97: 512–523. 16166562 10.1161/01.RES.0000182903.16652.d7

[13] Armulik A, Genove G, Mae M, Nisancioglu MH, Wallgard E, et al (2010) Pericytes regulate the blood-brain barrier. Nature 468: 557–561. 20944627 10.1038/nature09522

[14] Thompson RD, Noble KE, Larbi KY, Dewar A, Duncan GS, et al (2001) Platelet-endothelial cell adhesion molecule-1 (PECAM-1)-deficient mice demonstrate a transient and cytokine-specific role for PECAM-1 in leukocyte migration through the perivascular basement membrane. Blood 97: 1854–1860. 11238129 10.1182/blood.v97.6.1854

[15] Conlan JW, North RJ (1994) Neutrophils are essential for early anti-Listeria defense in the liver, but not in the spleen or peritoneal cavity, as revealed by a granulocyte-depleting monoclonal antibody. J Exp Med 179: 259–268. 8270870 10.1084/jem.179.1.259 PMC2191333

[16] Horley KJ, Carpenito C, Baker B, Takei F (1989) Molecular cloning of murine intercellular adhesion molecule (ICAM-1). EMBO J 8: 2889–2896. 2573511 10.1002/j.1460-2075.1989.tb08437.x PMC401350

[17] Dangerfield J, Larbi KY, Huang MT, Dewar A, Nourshargh S (2002) PECAM-1 (CD31) homophilic interaction up-regulates alpha6beta1 on transmigrated neutrophils in vivo and plays a functional role in the ability of alpha6 integrins to mediate leukocyte migration through the perivascular basement membrane. J Exp Med 196: 1201–1211. 12417630 10.1084/jem.20020324 PMC2194111

[18] Nakamura K, Fujiwara H, Higuchi T, Honda T, Nakayama T, et al (1997) Integrin alpha6 is involved in follicular growth in mice. Biochem Biophys Res Commun 235: 524–528. 9207189 10.1006/bbrc.1997.6825

[19] Fadok VA, Savill JS, Haslett C, Bratton DL, Doherty DE, et al (1992) Different populations of macrophages use either the vitronectin receptor or the phosphatidylserine receptor to recognize and remove apoptotic cells. J Immunol 149: 4029–4035. 1281199

[20] Wang S, Dangerfield JP, Young RE, Nourshargh S (2005) PECAM-1, alpha6 integrins and neutrophil elastase cooperate in mediating neutrophil transmigration. J Cell Sci 118: 2067–2076. 15840647 10.1242/jcs.02340

[21] Scheef EA, Sorenson CM, Sheibani N (2009) Attenuation of proliferation and migration of retinal pericytes in the absence of thrombospondin-1. Am J Physiol Cell Physiol 296: C724–734. 19193867 10.1152/ajpcell.00409.2008 PMC2670648

[22] Kim T, Hempstead BL (2009) NRH2 is a trafficking switch to regulate sortilin localization and permit proneurotrophin-induced cell death. EMBO J 28: 1612–1623. 19407813 10.1038/emboj.2009.118 PMC2693153

[23] Arthur WT, Burridge K (2001) RhoA inactivation by p190RhoGAP regulates cell spreading and migration by promoting membrane protrusion and polarity. Mol Biol Cell 12: 2711–2720. 11553710 10.1091/mbc.12.9.2711 PMC59706

[24] McMurdo L, Stephenson AH, Baldassare JJ, Sprague RS, Lonigro AJ (1998) Biosynthesis of sulfidopeptide leukotrienes via the transfer of leukotriene A4 from polymorphonuclear cells to bovine retinal pericytes. J Pharmacol Exp Ther 285: 1255–1259. 9618430

[25] Bainton DF, Miller LJ, Kishimoto TK, Springer TA (1987) Leukocyte adhesion receptors are stored in peroxidase-negative granules of human neutrophils. J Exp Med 166: 1641–1653. 2824654 10.1084/jem.166.6.1641 PMC2188781

[26] van Kooyk Y, Figdor CG (2000) Avidity regulation of integrins: the driving force in leukocyte adhesion. Curr Opin Cell Biol 12: 542–547. 10978887 10.1016/s0955-0674(00)00129-0

[27] Kim Y, Chang S (2004) Modulation of actomyosin contractility by myosin light chain phosphorylation/dephosphorylation through Rho GTPases signaling specifies axon formation in neurons. Biochem Biophys Res Commun 318: 579–587. 15120639 10.1016/j.bbrc.2004.04.068

[28] Schumacher C, Clark-Lewis I, Baggiolini M, Moser B (1992) High- and low-affinity binding of GRO alpha and neutrophil-activating peptide 2 to interleukin 8 receptors on human neutrophils. Proc Natl Acad Sci U S A 89: 10542–10546. 1438244 10.1073/pnas.89.21.10542 PMC50375

[29] Altieri DC (1991) Occupancy of CD11b/CD18 (Mac-1) divalent ion binding site(s) induces leukocyte adhesion. J Immunol 147: 1891–1898. 1890307

[30] Helluin O, Chan C, Vilaire G, Mousa S, DeGrado WF, et al (2000) The activation state of alphavbeta 3 regulates platelet and lymphocyte adhesion to intact and thrombin-cleaved osteopontin. J Biol Chem 275: 18337–18343. 10751402 10.1074/jbc.M001529200

[31] Kimura K, Ito M, Amano M, Chihara K, Fukata Y, et al (1996) Regulation of myosin phosphatase by Rho and Rho-associated kinase (Rho-kinase). Science 273: 245–248. 8662509 10.1126/science.273.5272.245

[32] Ridley AJ, Hall A (1992) The small GTP-binding protein rho regulates the assembly of focal adhesions and actin stress fibers in response to growth factors. Cell 70: 389–399. 1643657 10.1016/0092-8674(92)90163-7

[33] Dong JM, Leung T, Manser E, Lim L (1998) cAMP-induced morphological changes are counteracted by the activated RhoA small GTPase and the Rho kinase ROKalpha. J Biol Chem 273: 22554–22562. 9712882 10.1074/jbc.273.35.22554

[34] Seasholtz TM, Majumdar M, Kaplan DD, Brown JH (1999) Rho and Rho kinase mediate thrombin-stimulated vascular smooth muscle cell DNA synthesis and migration. Circ Res 84: 1186–1193. 10347093 10.1161/01.res.84.10.1186

[35] Lang P, Gesbert F, Delespine-Carmagnat M, Stancou R, Pouchelet M, et al (1996) Protein kinase A phosphorylation of RhoA mediates the morphological and functional effects of cyclic AMP in cytotoxic lymphocytes. EMBO J 15: 510–519. 8599934 PMC449969

[36] Ishizaki T, Uehata M, Tamechika I, Keel J, Nonomura K, et al (2000) Pharmacological properties of Y-27632, a specific inhibitor of rho-associated kinases. Mol Pharmacol 57: 976–983. 10779382

[37] Allingham JS, Smith R, Rayment I (2005) The structural basis of blebbistatin inhibition and specificity for myosin II. Nat Struct Mol Biol 12: 378–379. 15750603 10.1038/nsmb908

[38] Feng J, Ito M, Ichikawa K, Isaka N, Nishikawa M, et al (1999) Inhibitory phosphorylation site for Rho-associated kinase on smooth muscle myosin phosphatase. J Biol Chem 274: 37385–37390. 10601309 10.1074/jbc.274.52.37385

[39] Mitra P, Keese CR, Giaever I (1991) Electric measurements can be used to monitor the attachment and spreading of cells in tissue culture. Biotechniques 11: 504–510. 1793585

[40] Wegener J, Keese CR, Giaever I (2000) Electric cell-substrate impedance sensing (ECIS) as a noninvasive means to monitor the kinetics of cell spreading to artificial surfaces. Experimental Cell Research 259: 158–166. 10942588 10.1006/excr.2000.4919

[41] De Backer D, Biston P, Devriendt J, Madl C, Chochrad D, et al (2010) Comparison of dopamine and norepinephrine in the treatment of shock. N Engl J Med 362: 779–789. 20200382 10.1056/NEJMoa0907118

[42] Ward RM (1984) Pharmacology of tolazoline. Clin Perinatol 11: 703–713. 6386273

[43] Voisin MB, Woodfin A, Nourshargh S (2009) Monocytes and neutrophils exhibit both distinct and common mechanisms in penetrating the vascular basement membrane in vivo. Arterioscler Thromb Vasc Biol 29: 1193–1199. 19498176 10.1161/ATVBAHA.109.187450 PMC2712455

[44] Saunders WB, Bohnsack BL, Faske JB, Anthis NJ, Bayless KJ, et al (2006) Coregulation of vascular tube stabilization by endothelial cell TIMP-2 and pericyte TIMP-3. J Cell Biol 175: 179–191. 17030988 10.1083/jcb.200603176 PMC2064509

[45] Kalluri R (2003) Basement membranes: structure, assembly and role in tumour angiogenesis. Nat Rev Cancer 3: 422–433. 12778132 10.1038/nrc1094

[46] Puetz S, Lubomirov LT, Pfitzer G (2009) Regulation of smooth muscle contraction by small GTPases. Physiology (Bethesda) 24: 342–356. 19996365 10.1152/physiol.00023.2009

[47] Hixenbaugh EA, Goeckeler ZM, Papaiya NN, Wysolmerski RB, Silverstein SC, et al (1997) Stimulated neutrophils induce myosin light chain phosphorylation and isometric tension in endothelial cells. Am J Physiol 273: H981–988. 9277518 10.1152/ajpheart.1997.273.2.H981

[48] Saito H, Minamiya Y, Kitamura M, Saito S, Enomoto K, et al (1998) Endothelial myosin light chain kinase regulates neutrophil migration across human umbilical vein endothelial cell monolayer. J Immunol 161: 1533–1540. 9686621

[49] Gonul E, Duz B, Kahraman S, Kayali H, Kubar A, et al (2002) Early pericyte response to brain hypoxia in cats: an ultrastructural study. Microvasc Res 64: 116–119. 12074637 10.1006/mvre.2002.2413

[50] Peppiatt CM, Howarth C, Mobbs P, Attwell D (2006) Bidirectional control of CNS capillary diameter by pericytes. Nature 443: 700–704. 17036005 10.1038/nature05193 PMC1761848

[51] Cavaillon JM, Duff G (1999) Cytokines and the cellular mechanism of inflammation. In: The cytokine network and immune functions. (ed. Theze, J.) 251–261. Oxford: Oxford University Press.

[52] Stratman AN, Davis GE (2011) Endothelial cell-pericyte interactions stimulate basement membrane matrix assembly: influence on vascular tube remodeling, maturation, and stabilization. Microsc Microanal 18: 68–80. 22166617 10.1017/S1431927611012402 PMC3919655

[53] Stratman AN, Schwindt AE, Malotte KM, Davis GE (2010) Endothelial-derived PDGF-BB and HB-EGF coordinately regulate pericyte recruitment during vasculogenic tube assembly and stabilization. Blood 116: 4720–4730. 20739660 10.1182/blood-2010-05-286872 PMC2996127

[54] Proebstl D, Voisin MB, Woodfin A, Whiteford J, D’Acquisto F, et al (2012) Pericytes support neutrophil subendothelial cell crawling and breaching of venular walls in vivo. J Exp Med 209(6): 1219–34. 22615129 10.1084/jem.20111622 PMC3371725

