# Supplementary figures and images for: Pericytes Regulate Vascular Basement Membrane Remodeling and Govern Neutrophil Extravasation during Inflammation
Source: PLoS One. 2012 Sep 21;7(9):e45499. doi: 10.1371/journal.pone.0045499 (PMC3448630; doi:10.1371/journal.pone.0045499)

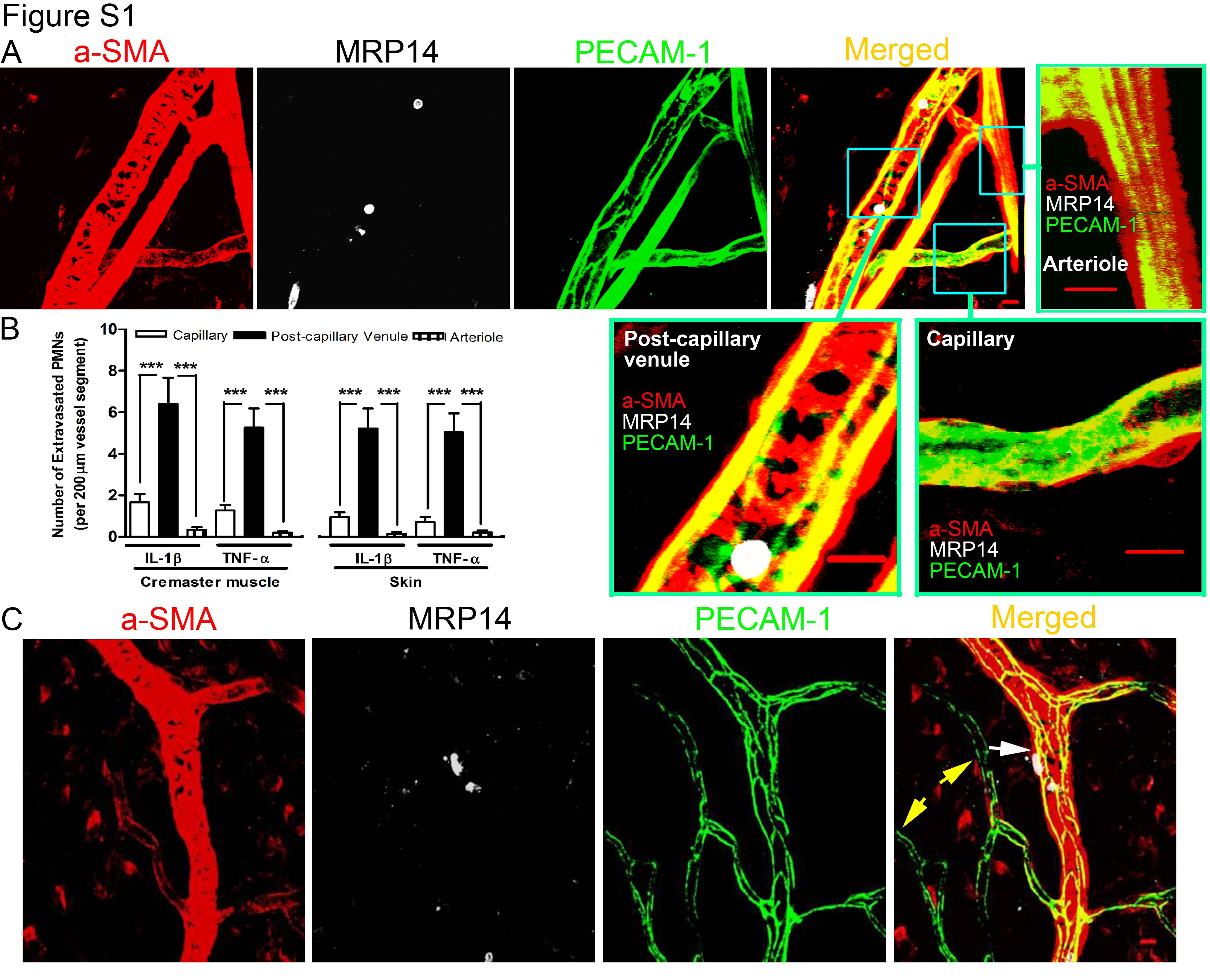

Supplement: Figure S1 — Expression of α-SMA in microvasculature and occurrence of PMN extravasation. (A) Representative images show different types of vessel segments, among which PMN extravasation occurred primarily at the post-capillary venule. Mouse cremaster muscles were injected with IL-1β. Two hours later, tissues were collected, immuno-stained for α-SMA, MRP14 and PECAM-1, and imaged in 3D. The areas outlined in the merged image panel are shown magnified in the corresponding panels as indicated, so as to reveal the expression of α-SMA and PECAM-1 in the arteriole, capillary and venule. Note: due to the relatively higher expression of α-SMA in the arteriole, the arteriole image intensity was saturated. Bar = 10 µm. (B) Quantitation of PMNs extravasated from capillaries, post-capillary venules or arterioles in mouse cremaster muscles or skin which were stimulated by either IL-1α or TNF-α. Ten vessel segments/mouse and four mice/group were analyzed. ANOVA plus Neuman-Keuls multiple comparisons were performed as indicated. *** P<0.001. (C) TNF-α-injected mouse cremaster muscles were immuno-stained for smooth muscle actin, PECAM-1 and MRP14 and imaged in 3D. α-SMA staining in capillaries was faint and even negative (indicated by yellow arrows in the merged image) but strong in post-capillary venules (indicated by a white solid arrow). PMN extravasation took place at the post-capillary venule. Bar = 10 µm. (TIF) [file pone.0045499.s001.tif]

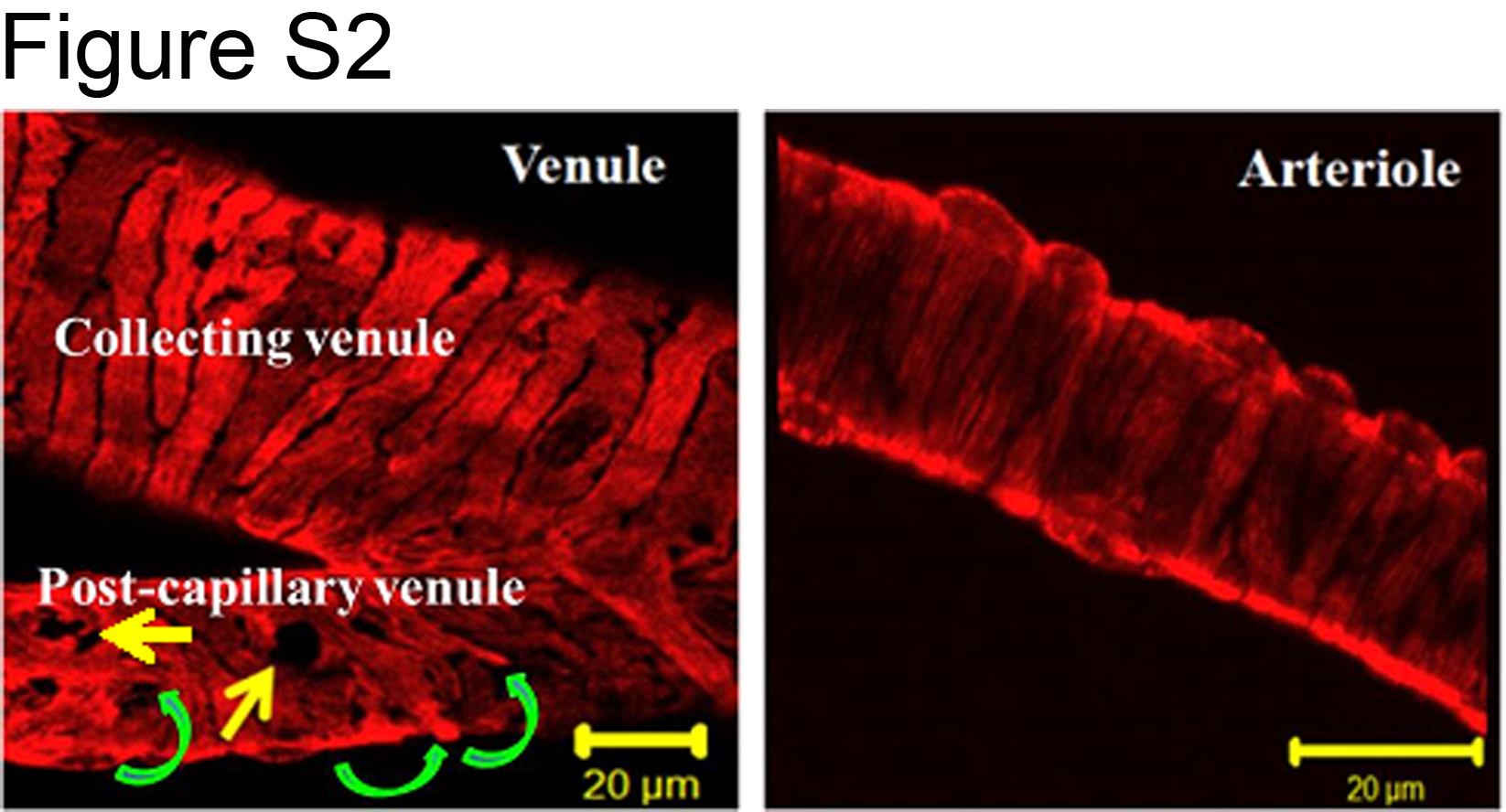

Supplement: Figure S2 — α-SMA staining in an arteriole, a post-capillary venule, and a collecting venule of mouse cremaster muscles. Resting tissues were immuno-stained for α-SMA, imaged in 3D and displayed at high magnification. In the arteriole, α-SMA positive cells (i.e. smooth muscle cells) regularly surrounded the vessel like compact coils. However, α-SMA positive cells intricately presented in the post-capillary venule and extended a number of longitudinal and circumferential processes (indicated by green arc arrows), exhibiting a typical pericyte phenotype. Gaps between adjacent cells were marked by yellow arrows. In the collecting venule, α-SMA positive cells (i.e. muscle cells) regularly coiled around the vessel wall. (TIF) [file pone.0045499.s002.tif]

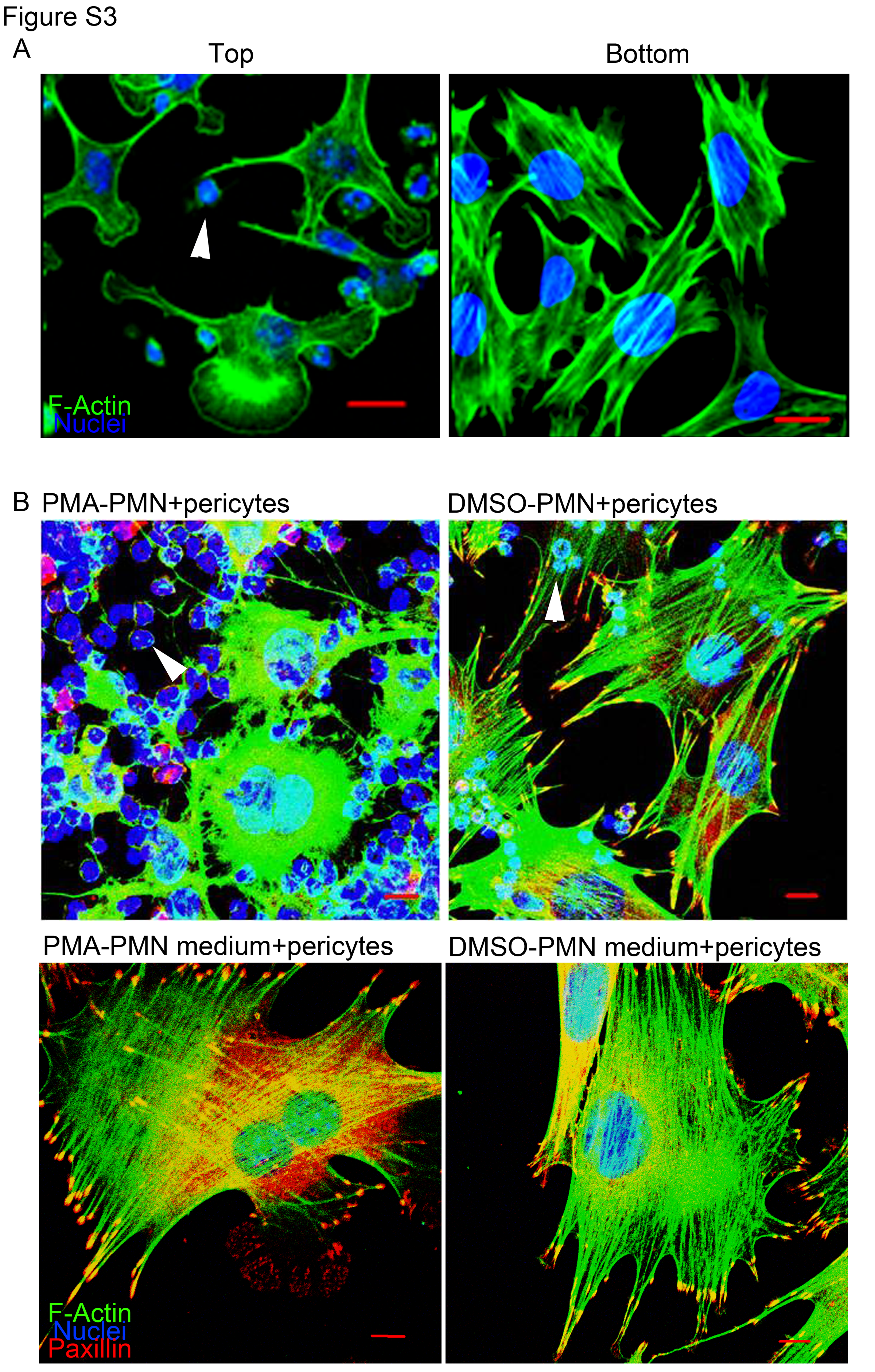

Supplement: Figure S3 — Direct contacts with PMA-activated PMNs lead to pericyte relaxation. To further confirm that pericyte responses to PMA-treated PMNs were mediated by direct cell-cell contacts between PMNs and pericytes rather than by the residual PMA in the culture medium, we developed two experimental strategies as described below. (A) In serum-free medium containing TNF-α (15 ng/ml), mouse primary pericytes were grown on two sides of a FN-coated coverslip placed in the well of a 24 well plate overnight. After being extensively washed, PMA-treated PMNs were added on the pericyte layer growing on the top surface of coverslips for one hour. Cells were fixed and stained for F-actin and nuclei. Pericytes on the top surface (left image) exhibited large lamellipodia and lost actin stress fibers, compared to the cells growing on the bottom surface (right image). (B) In serum-free medium containing TNF-α (15 ng/ml), mouse primary pericytes were grown on FN-coated coverslips placed in the well of a 24 well plate overnight. After being extensively washed, PMA- or DMSO-treated PMNs were added. One hour later, the conditioned medium was collected from these wells and any contaminating cells (probably including some pericytes and PMNs) were removed by centrifugation. These conditioned media were added to another batch of pericytes growing on FN-coated coverslips. Cells were fixed and stained for F-actin, paxillin and nuclei. Exposure to PMA-treated (top left image) rather than DMSO-treated PMNs (top right image) led to loss of actin stress fibers and focal adhesions in pericytes. However, conditioned medium collected from the wells, in which either PMA-treated (bottom left image) or DMSO-treated PMNs (bottom right image) had interacted with pericytes, had no influence on the morphology and cytoskeleton in the second batch of pericytes. Arrowheads indicate PMNs. Bar = 10 µm. Data shown in (A) and (B) suggest that direct contacts with PMA-activated PMNs mediate pericyte relaxation. (TIF) [file pone.0045499.s003.tif]

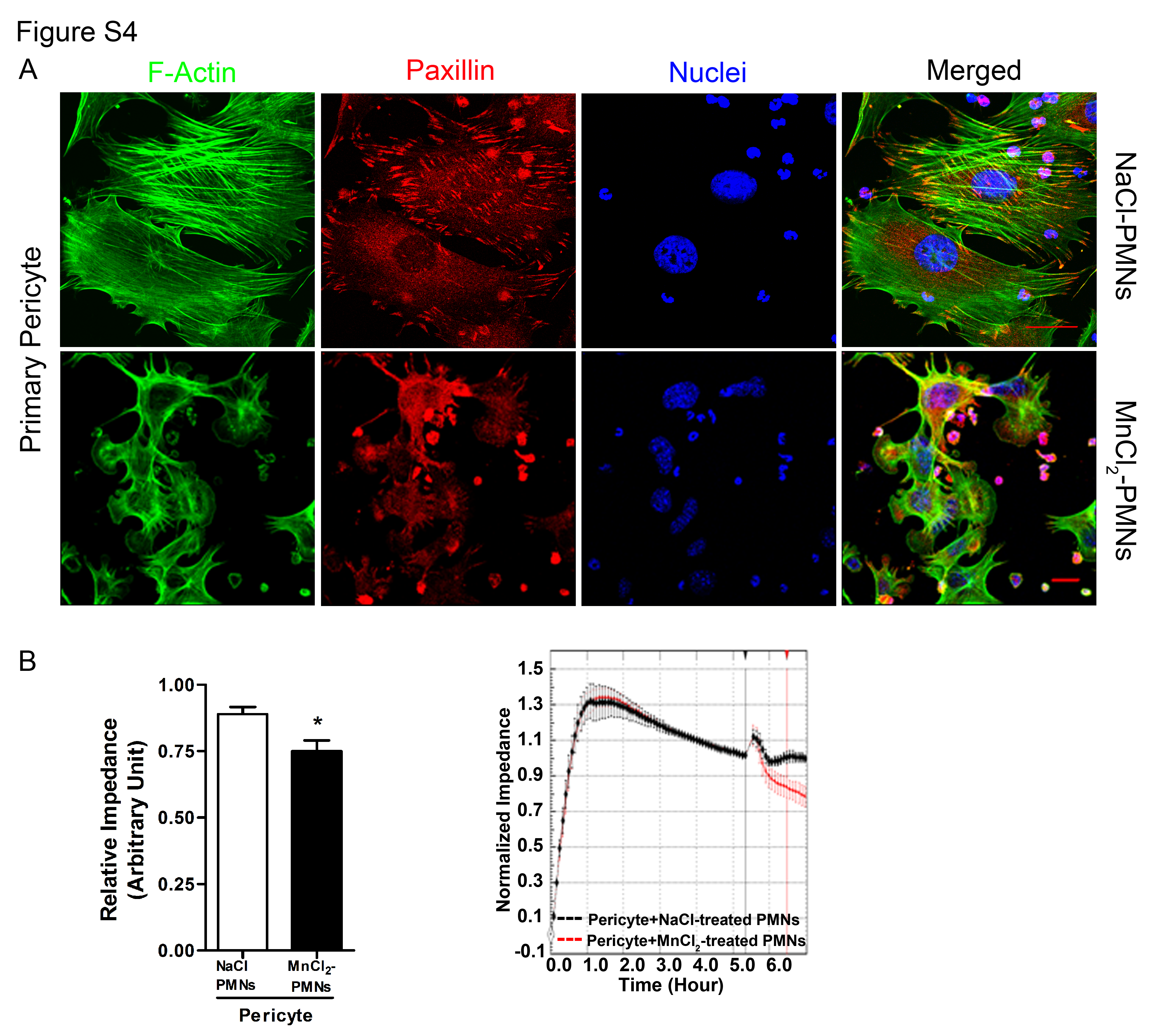

Supplement: Figure S4 — Engagement of MnCl2-activated PMNs leads to pericyte relaxation and increases permeability of pericyte monolayers. (A) TNF-α-induced pericytes were grown on FN-coated coverslips. NaCl- or MnCl2-treated PMNs that had been extensively washed after stimulation were added for 1 hour. Cells were fixed and stained for nuclei, F-actin and paxillin. MnCl2-treated PMNs induced partial loss of actin stress fibers and focal adhesions in pericytes. (B) TNF-α-induced mouse primary pericytes were seeded on the wells of a RTCA analyzer for 3 to 5 hours to make a confluent cell monolayer. NaCl- or MnCl2-treated PMNs were respectively added to each well for one more hour. The impedance of a pericyte monolayer was recorded in time-lapse. As demonstrated by the representative experimental curves in the right graph, the relative impedance at 1 hour after adding NaCl- or MnCl2-treated PMNs (indicated by red vertical line) was normalized to that when PMNs were added (indicated by black vertical line). Exposure to MnCl2-treated PMNs caused a decrease of the impedance in pericyte monolayers (left bar graph). T test, *P<0.05. (TIF) [file pone.0045499.s004.tif]

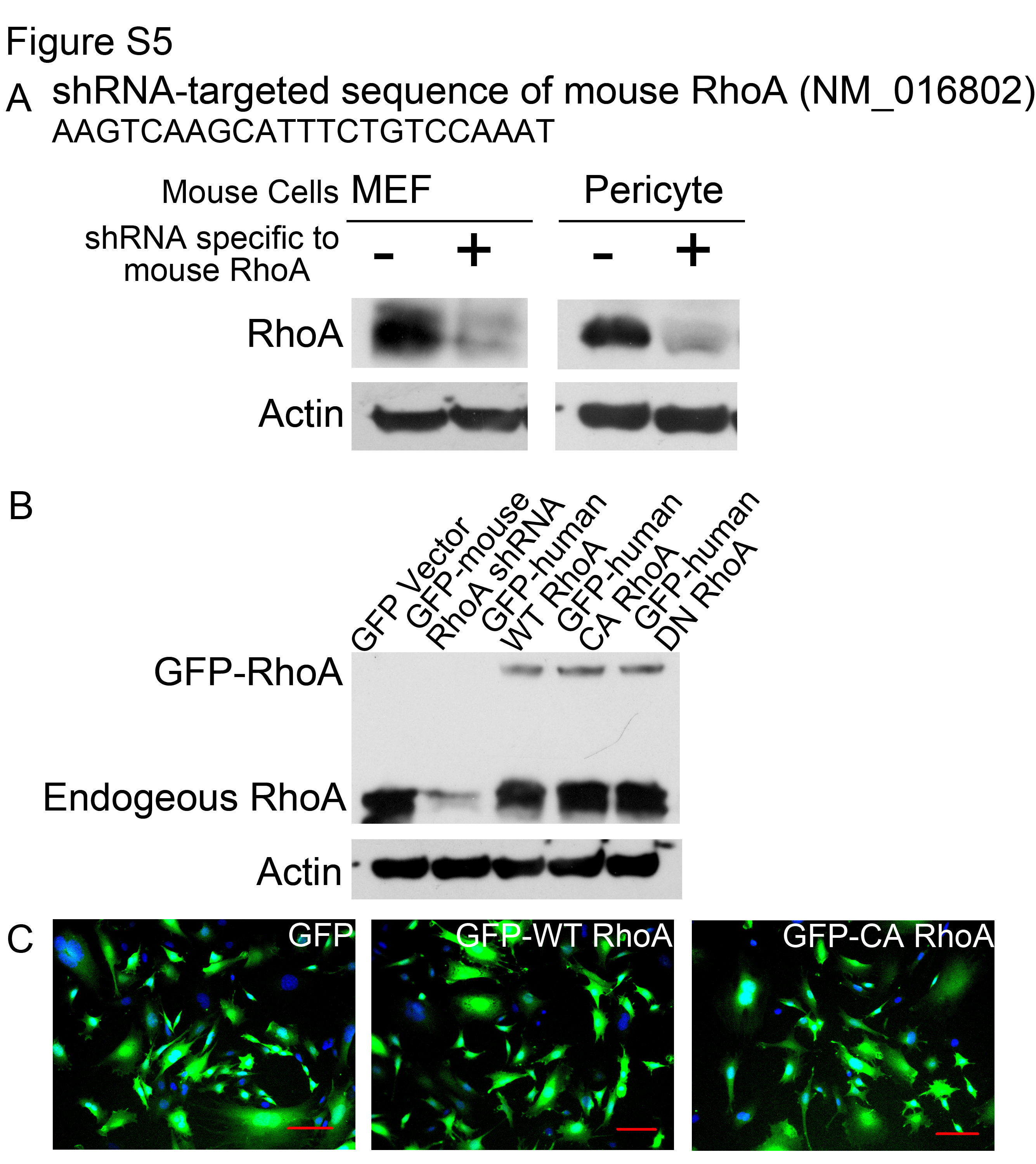

Supplement: Figure S5 — Sequence of shRNA specific to mouse RhoA and expression of exogenous GFP-targeted RhoA. To analyze the role of RhoA in pericytes interacting with PMNs, we infected mouse primary pericytes with lentivirus bearing a GFP reporter and short-hairpin RNA (shRNA) to knock down RhoA in mouse pericytes. In some cases, we expressed exogenous GFP-tagged wild type (WT), constitutively active (CA) Q63L or dominant negative (DN) T19N RhoA in mouse primary pericytes. This allowed us to distinguish RhoA in pericytes from RhoA in PMNs when investigating pericyte interactions with PMNs. (A) ShRNA-targeted sequence of mouse RhoA is displayed, as well as the expression levels of endogenous RhoA in mouse MEF cells and primary pericytes as revealed by western blots in control cells or following knockdown by the shRNA. (B) Expression of exogenous GFP-tagged human RhoA in mouse primary pericytes was revealed by western blots. (C) Representative images showed expression of GFP or GFP-tagged RhoA in mouse primary pericytes. Nuclei were displayed in blue. Approximately 90% GFP positive cells were achieved. Bar = 50 µm. (TIF) [file pone.0045499.s005.tif]

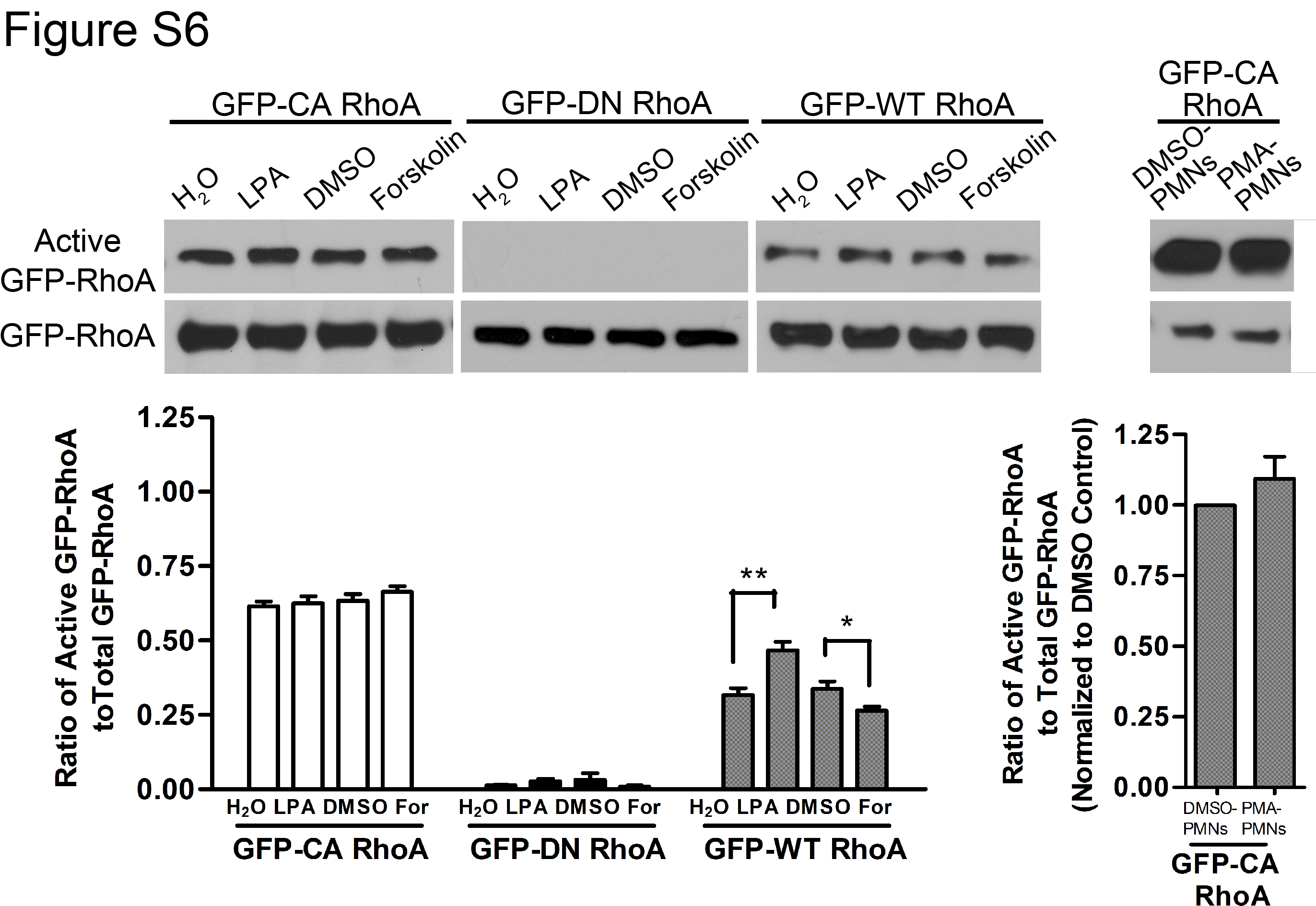

Supplement: Figure S6 — Assay of GFP-tagged RhoA in pericytes after exposure to different chemicals or engagement with PMNs. Pericytes expressing GFP-tagged CA, DN or WT human RhoA were starved in serum-free medium supplied with TNF-α overnight and exposed to the indicated reagents or to DMSO-treated or PMA-treated PMNs. Activity of GFP-tagged RhoA was detected (n = 7 for pericytes treated with chemicals and n = 3 for pericytes interacting with PMNs. t test. * P<0.05 and ** P<0.01 as indicated). (TIF) [file pone.0045499.s006.tif]

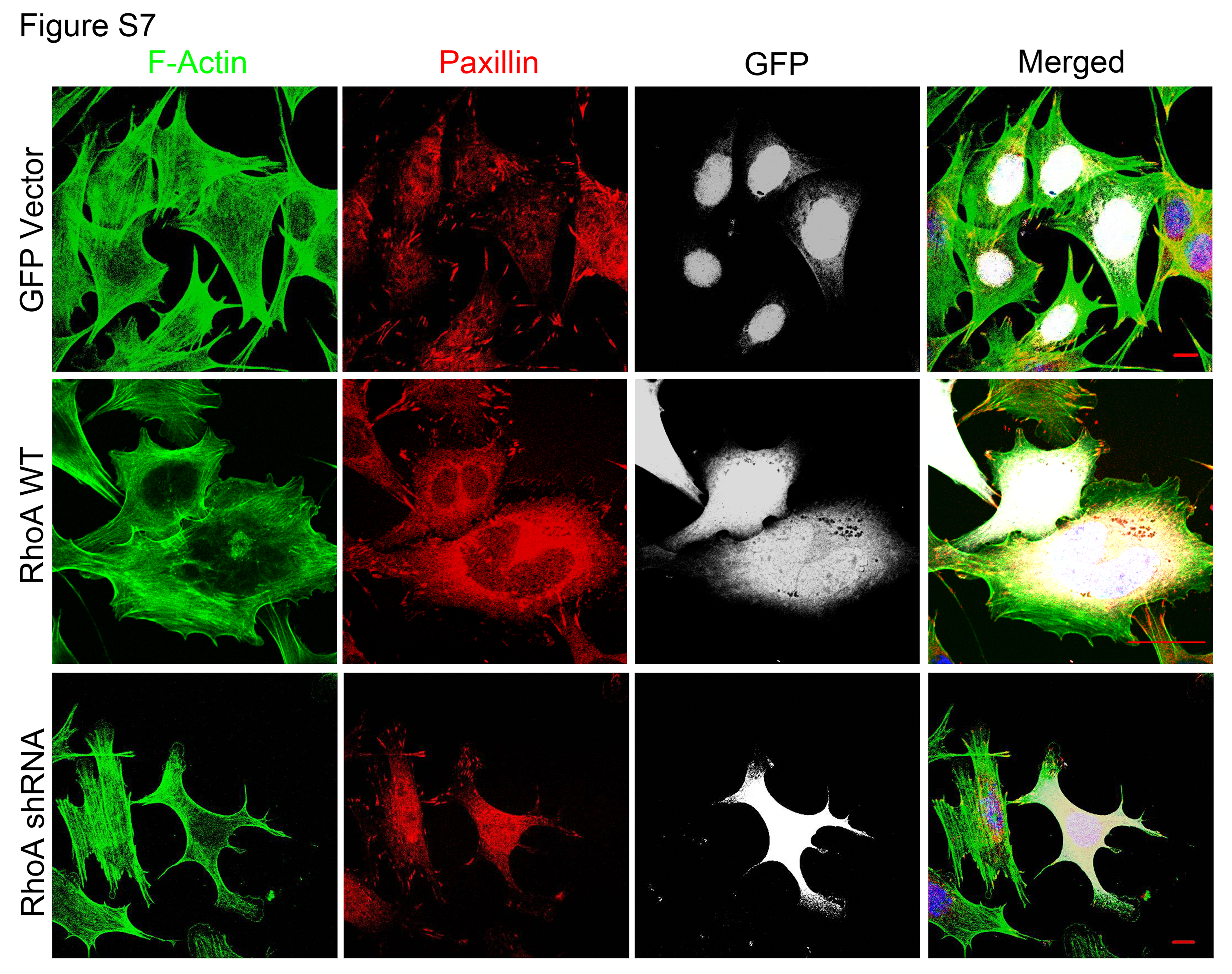

Supplement: Figure S7 — Knocking down RhoA expression leads to a relaxation phenotype in pericytes. Mouse pericytes were infected with lentivirus carrying GFP empty vector, or GFP-tagged human WT RhoA or shRNA specific to mouse RhoA. Cells were seeded on FN-coated coverslips and stained for F-actin (green), paxillin (red) and nuclei (blue). Samples were imaged and GFP was displayed in white. Compared to GFP-negative cells or those expressing GFP empty vector or GFP-tagged WT RhoA, pericytes bearing shRNA for RhoA extended protrusions and lost stress fibers and focal adhesions. Bar = 10 µm. (TIF) [file pone.0045499.s007.tif]

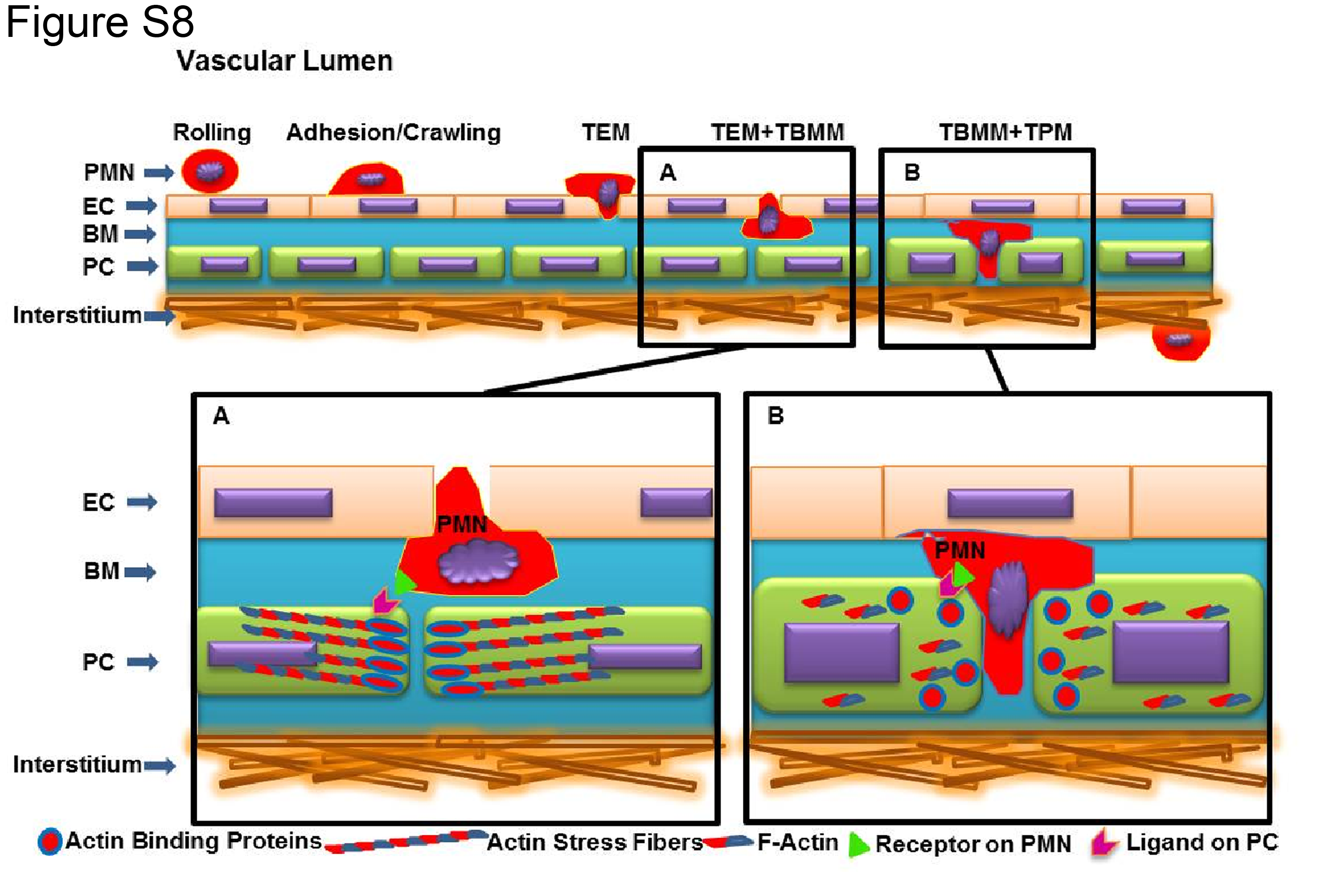

Supplement: Figure S8 — A diagram illustrating the interactions of PMNs migrating across a venule wall. The top diagram demonstrates the structure of a venule wall, illustrating that the endothelium contacts the vascular basement membrane with its abluminal surface, whereas the pericyte sheath is embedded in this basement membrane. In some cases pericytes may also establish interactions with endothelial cells via extended processes (not shown). The diagram also shows PMNs at different stages of migration. The boxed areas (A and B) in the top diagram are displayed at higher magnification in the corresponding bottom panels. PMN = Polymorphonuclear neutrophil; EC = endothelial cell; BM = Basement membrane; PC = Pericyte; TEM = Trans-endothelial migration; TBMM = Trans-basement membrane migration; TPM = Trans-pericyte migration. Box A shows a transmigrating PMN that has broken through the endothelium but is separated from the pericyte sheath by the vascular BM. In this situation, pericytes still maintain most of their actin bundles and focal adhesions which support the binding of pericytes to the BM. Diffusible inflammatory mediators (e.g. nitric oxide or complement C3a/5a) may relax pericytes to a certain extent, but this is not indicated in the diagram. At the stage shown in box B, the transmigrating PMN has penetrated the BM and established direct contacts with the pericyte sheath. Direct engagement with the migrating PMN elicits signals to induce pericyte relaxation disassembling actin stress fibers. Relaxation of pericytes expands the gaps between them and thins (or opens) the BM at LERs which align with pericyte gaps, thereby facilitating PMN transmigration. (TIF) [file pone.0045499.s008.tif]
